# Optimal dose and type of exercise to reduce pain, anxiety and increase quality of life in patients with fibromyalgia. A systematic review with meta-analysis

**DOI:** 10.3389/fphys.2023.1170621

**Published:** 2023-04-12

**Authors:** Daniel Rodríguez-Almagro, María Del Moral-García, María del Carmen López-Ruiz, Irene Cortés-Pérez, Esteban Obrero-Gaitán, Rafael Lomas-Vega

**Affiliations:** ^1^ Department of Nursing, Physiotherapy and Medicine, University of Almería, Almería, Spain; ^2^ Department of Health Sciences, University of Jaén, Jaén, Spain

**Keywords:** fibromyalgia, exercise therapy, pain, quality of life, anxiety, women, disability, recommended dose

## Abstract

The aim of our meta-analysis was to compile the available evidence to evaluate the effect of physical exercise-based therapy (PEBT) on pain, impact of the disease, quality of life (QoL) and anxiety in patients with fibromyalgia syndrome (FMS), to determine the effect of different modes of physical exercise-based therapy, and the most effective dose of physical exercise-based therapy for improving each outcome. A systematic review and meta-analysis was carried out. The PubMed (MEDLINE), SCOPUS, Web of Science, CINAHL Complete and Physiotherapy Evidence Database (PEDro) databases were searched up to November 2022. Randomized controlled trials (RCTs) comparing the effects of physical exercise-based therapy and other treatments on pain, the impact of the disease, QoL and/or anxiety in patients with FMS were included. The standardized mean difference (SMD) and a 95% CI were estimated for all the outcome measures using random effect models. Three reviewers independently extracted data and assessed the risk of bias using the PEDro scale. Sixty-eight RCTs involving 5,474 participants were included. Selection, detection and performance biases were the most identified. In comparison to other therapies, at immediate assessment, physical exercise-based therapy was effective at improving pain [SMD-0.62 (95%CI, −0.78 to −0.46)], the impact of the disease [SMD-0.52 (95%CI, −0.67 to −0.36)], the physical [SMD 0.51 (95%CI, 0.33 to 0.69)] and mental dimensions of QoL [SMD 0.48 (95%CI, 0.29 to 0.67)], and the anxiety [SMD-0.36 (95%CI, −0.49 to −0.25)]. The most effective dose of physical exercise-based therapy for reducing pain was 21–40 sessions [SMD-0.83 (95%CI, 1.1–−0.56)], 3 sessions/week [SMD-0.82 (95%CI, −1.2–−0.48)] and 61–90 min per session [SMD-1.08 (95%CI, −1.55–−0.62)]. The effect of PEBT on pain reduction was maintained up to 12 weeks [SMD-0.74 (95%CI, −1.03–−0.45)]. Among patients with FMS, PEBT (including circuit-based exercises or exercise movement techniques) is effective at reducing pain, the impact of the disease and anxiety as well as increasing QoL.

**Systematic Review Registration:** PROSPERO https://www.crd.york.ac.uk/PROSPERO/, identifier CRD42021232013.

## 1 Introduction

Fibromyalgia syndrome (FMS) is a chronic musculoskeletal disorder mainly characterized by impaired pain processing, resulting in generalized, diffuse and non-inflammatory pain in different body localizations (Galvez-Sánchez and Reyes del Paso, 2020; Sarzi-Puttini et al., 2020). In addition to pain, other disabling symptoms of FMS include movement restrictions, fatigue (Gota, 2022), balance disorders (Peinado-Rubia et al., 2020; Núñez-Fuentes et al., 2021), mood disorders [such as anxiety, depression or low self-esteem (Galvez-Sánchez et al., 2019)], sleep disorders (Frange et al., 2014) and sexual dysfunctions (Ricoy-Cano et al., 2021). FMS is a highly prevalent disorder–it affects approximately 2.4% of the world’s population (Queiroz, 2013). Moreover, it has been found to be more common among women (the ratio of women to men is 3:1) and middle-aged people (approximately 30–50 years of age (Queiroz, 2013; Häuser et al., 2015)). FMS causes a high socioeconomic burden on the healthcare system (Skaer, 2014). It is estimated to cost €7256–7900 per patient each year in developed countries; furthermore, it leads to high rates of absenteeism, unemployment, and early retirement as well as a higher number of days off work (Schaefer et al., 2011). These costs are mainly due to medical visits, specialized consultations, diagnostic tests, medicines and complementary therapies to provide psychological support (Feliu-Soler et al., 2016).

Despite the prevalence of this health problem, its causes are still unknown, and the pathophysiology of FMS is not entirely clear (Schmidt-Wilcke and Diers, 2017). Early etiological theories were mainly based on psychogenic factors, as no physical signs were found to justify the pain in these patients (Bair and Krebs, 2020). Over time, advances in research have cast doubt on these hypotheses, suggesting that FMS may be caused by a process of central sensitization and a mismatch in pain processing (Staud et al., 2007; Goubert et al., 2017; Bair and Krebs, 2020; Oliva et al., 2022). Various neuroimaging tests have revealed alterations in the central nervous system (CNS), such as morphological changes in the brain regions in charge of processing nociceptive stimuli, an increase in nervous activity in these areas and an imbalance in the concentration of related neurotransmitters (Giesecke et al., 2004; Napadow and Harris, 2014; Schmidt-Wilcke and Diers, 2017; Bair and Krebs, 2020), which could lead to an exacerbation of painful sensations and a decrease in endogenous pain inhibition systems (O’Brien et al., 2018; Bair and Krebs, 2020). Although the specific cause of FMS is not yet known, numerous risk factors have been identified, such as previous medical pathologies, poor quality of life (QoL), sedentary lifestyle, depression, hypochondria, childhood problems or history of abuse (Creed, 2020).

Due to the considerable heterogeneity of FMS symptomatology, there is no single treatment for all patients (Häuser et al., 2015). According to a meta-analysis by Nüesch et al., 2013, FMS requires multidisciplinary management that includes both pharmacological and non-pharmacological measures (Nüesch et al., 2013). The measures applied should mainly focus on symptom management and increasing functionality and QoL (Bair and Krebs, 2020). According to the latest guidelines proposed by the European League against Rheumatism (EULAR) for the management of FMS, the therapeutic pillars should be cognitive behavioral therapy and graded and paced physical exercise (Macfarlane et al., 2017). Physical exercise-based therapy (PEBT) includes a large variety of aerobic, resistance, strength, balance, and proprioceptive exercises that can help to reduce pain and muscle debility in these patients and could increase their QoL. Some of the main advantages of PEBT are that it is an active and ludic therapy that can be performed in groups and that can increase social support between patients with FMS. Pilates (Franco et al., 2023), dance (Murillo-Garcia et al., 2022), yoga (Allsop et al., 2022), tai chi (Cheng et al., 2019), circuit training (aerobic, strength and multicomponent) (Vilarino et al., 2021; Araya-Quintanilla et al., 2022; Estrada-Marcén et al., 2023), body awareness therapy (Bravo et al., 2019), and videogames (Cortés-Pérez et al., 2021) are the most common forms to peform PEBT in patients with FMS; the effects of these forms of therapy have been widely assessed in the scientific literature, and promising results have been obtained.

In recent years, some reviews have assessed the effect of different types of PEBT to improve more common symptoms in FMS. An interesting and common finding in these reviews is that patients undergoing PEBT do not report adverse events, thus indicating that PEBT is a safe therapy to use in FMS (Bidonde et al., 2014a). Although the results presented in these works generally support the use of PEBT, they are difficult to synthesize, as each review assessed one specific type of PEBT, such as Tai Chi (Cheng et al., 2019), flexibility exercise training (Kim et al., 2019), aquatic exercise (Bidonde et al., 2014b) or exergames using virtual reality devices (Cortés-Pérez et al., 2021). In addition, other reviews assessed different exercise approaches, but the number of studies included was low, making it difficult to generalize the findings (Sosa-Reina et al., 2017; Del-Moral-García et al., 2020; Estévez-López et al., 2021). To date, there have been no comprehensive reviews that assess the effect of different PEBT modalities and provide evidence about the correct doses for patients with FMS. Therefore, the primary objective of this systematic review and meta-analysis was to compile all the available evidence to assess the effect of PEBT on pain, disability impact, QoL and anxiety in patients with FMS. As a secondary objective, we aimed to determine the appropriate dose of PEBT to improve each outcome in FMS patients. Finally, we aimed to assess the effect of PEBT according to specific modalities of PEBT (circuit-based exercise or exercise-movement techniques).

## 2 Methods

### 2.1 Protocol and registration

The current systematic review and meta-analysis was conducted in accordance with the Preferred Reporting Items for Systematic Reviews and Meta-Analysis (PRISMA 2020 statement) (Page et al., 2021) and the Cochrane Handbook for Systematic Reviews of Interventions (Higgins and Thomas, 2020). In addition, the protocol of this review was previously registered in the International Prospective Register of Systematic Reviews (PROSPERO: CRD42021232013).

### 2.2 Literature search strategy

Two authors, independently, searched the PubMed (MEDLINE), Scopus, Web of Science (WOS), CINAHL Complete and Physiotherapy Evidence Database (PEDro) databases up to November 2022. Additional sources were searched, including previously published reviews, gray literature and expert documents. Boolean operators were used in the search and we do not use restrictions related with publication date and language. All searches were supervised by a third author who is an expert in bibliographic searches. The following Medical Subject Headings (MeSH) terms were used to search the PubMed (MEDLINE) database: (*fatigue syndrome, chronic[mh] OR fatigue syndrome, chronic[tiab] OR fibromyalgia[mh] OR fibromyalgia[tiab]*) *AND* (*exercise[mh] OR exercise[tiab] OR exercise therapy[mh] OR exercise therapy[tiab] OR physical exercise[tiab] OR physical activity[tiab] OR training[tiab]*) *AND* (*randomized controlled trial[publication type] OR randomized controlled trial[tiab] OR clinical trial[publication type] OR clinical trial[tiab] OR controlled clinical trial[publication type] OR controlled clinical trial[tiab]*) *NOT* (*systematic review[publication type] OR systematic review[tiab] OR meta-analysis[publication type] OR meta-analysis[tiab] OR review[publication type] OR review[tiab]*)*.* This search strategy was adapted to the other databases (Supplementary Table S1).

### 2.3 Inclusion and exclusion criteria

In accordance with the PICO framework, the inclusion criteria were as follows: 1) Population, patients diagnosed with FMS; 2) Intervention, PEBT; 3) Comparison, interventions other from PEBT, including usual care; and 4) Outcomes, pain, the impact of FMS, anxiety and physical/mental QoL. Additionally, we included randomized controlled trials (RCTs) and pilot RCTs that provided post-intervention quantitative data (n, mean and standard deviation of each group) of the outcomes of interest, thus enabling us to perform meta-analysis. The exclusion criteria were 1) studies whose population did not comprise exclusively FMS patients and 2) studies that reported quantitative data that were not suitable for meta-analysis.

### 2.4 Data extraction

Two authors, independently, analyzed the titles and abstracts of each reference retrieved. If a study was selected by one of the authors, it was examined in detail to determine its inclusion or exclusion and the corresponding reasons for extracting the data of interest for meta-analysis. Disagreements between the two reviewers were resolved by consulting a third reviewer. The data extracted of the articles selected were collected in a standardized form in Microsoft Excel. A third author was consulted in case of disagreements. The following data were extracted from each study: authorship and publication data, country and total sample size. From each group, we collected sample size, age (mean or range), body mass index (BMI) and gender. From the experimental intervention (PEBT) groups, we extracted the type of PEBT (exercise-based circuit or exercise movement techniques) and the protocol of application (weeks, sessions per week and minutes per session). From the comparison intervention groups, we extracted the type of intervention. Finally, regarding the outcomes of interest, we extracted the test employed in each study and the quantitative data (mean and standard deviation). If the means and standard deviations were not available, other types of statistical data (median, standard error or interquartile range) were collected so that they could be transformed and subsequently included in the current meta-analysis (Hozo et al., 2005; Higgins and Thomas, 2020).

### 2.5 Variables

The outcomes examined in this systematic review and meta-analysis were as follows: pain, the impact of FMS, QoL (physical and mental dimension), and anxiety experienced in patients with FMS. To assess these variables, we included quantitative data from validated tests that measure the same construct. So, for pain, we would include data from the Visual Analogue Scale (VAS), the Numeric Pain Rating Scale (NPRS) or pain dimension of the Fibromyalgia Impact Questionnaire (FIQ), among others. For impact of FMS, we would include studies that assessed it with FIQ; for QoL, questionnaires or scales that assessed it, such as SF-36 or EuroQoL-5D; and finally, for anxiety, questionnaires such as Beck Anxiety Inventory (BAI) or Hospital Anxiety and Depression Inventory (HADS), can be selected.

### 2.6 Quality assessment

The PEDro scale was used to assess the methodological quality and risk of bias of the studies included in the review. This scale is composed of 11 items that can be scored as “yes” (if the criteria are met) or “no” (if the criteria are not met) (Macedo et al., 2010). The total score ranged from 0 (very low methodological quality and high risk of bias) to 10 (high methodological quality and very low risk of bias). The PEDro scale categorizes methodological quality as “excellent” (10–9 points), “good” (eight to six points), “fair” (five to four points), and “poor” (3 points or less) (Cashin and McAuley, 2020).

To assess the quality of evidence in each meta-analysis, we used the Grading of Recommendations Assessment, Development, and Evaluation (GRADE) (Atkins et al., 2004). The quality of evidence is determined based on the following items: risk of bias in each study, inconsistency, indirect evidence, imprecision and risk of publication bias. All these items, except the risk of bias, were assessed using the GRADE checklist of Meader (Meader et al., 2014). Two authors, independently, participated in these assessments, and doubts were resolved by a third author.

### 2.7 Statistical analysis

Statistical analysis was performed with Comprehensive Meta-Analysis version 3.0 (Biostat, Englewood, NJ, United States) by two authors. Meta-analysis was only performed if at least two studies reported data for an outcome. The DerSimonian and Laird random effects analysis was employed (DerSimonian and Laird, 1986), and the effect size was calculated using Cohen’s standardized mean difference (SMD) and its 95% CI (Cohen J, 1977). Cohen’s SMD can be categorized into four levels: no effect (SMD = 0), small (SMD = 0.2), medium (SMD = 0.5) and large (SMD >0.8) (Faraone, 2008). In addition, when an outcome was assessed with the same test, the mean difference (MD) between groups was calculated to compare our results with the minimum clinically important difference (MCID) for the test. The results of each meta-analysis were shown in forest plots (Rücker and Schwarzer, 2020), and the risk of publication bias was assessed *via* visual analysis of the forest plot (a symmetric plot indicates a low risk of publication bias, and an asymmetric plot indicated a high risk of publication bias) (Sterne and Egger, 2001) and with Egger’s test (*p* < 0.1 indicates possible risk of publication) (Egger et al., 1997). Furthermore, the trim-and-fill calculation was used to estimate the adjusted SMD, taking into account any possible risk of publication bias (Duval and Tweedie, 2000). In accordance with Rothman’s recommendations for the effect size variation limit in the assessment of confounding bias, when the adjusted SMD varied by more than 10% from the original, raw pooled effect, the level of quality of evidence lowered by one level, even if the funnel plot was only slightly asymmetric (Rothman et al., 2008). Finally, the heterogeneity was assessed with the Q-test (*p* < 0.1 indicates risk of heterogeneity) and the degree of inconsistency (I^2^) (I^2^ <25% indicates low heterogeneity; I^2^ between 25%–50% indicates moderate heterogeneity, and I^2^ >50% indicates high heterogeneity) (Higgins et al., 2002; Higgins et al., 2003).

The contribution of each study to the overall pooled effect was assessed *via* sensitivity analysis using the leave-one-out method (Higgins and Thomas, 2020). In addition, multiple subgroup analyses were performed. First, a subgroup analysis was performed based on the specific PEBT modality employed: circuit-based exercise (aerobic, strength, flexibility, endurance exercises) *versus* other therapies, and exercise-movement techniques (Tai Chi, yoga, Pilates) *versus* other therapies. A second subgroup analysis was performed based on the follow-up duration: immediate (just after completion of therapy), short-term (12 weeks), medium-term (24 weeks) and long-term (48 weeks). In addition, to determine the optimal dose of PEBT for improvement of each outcome, subgroup analyses were performed based on 1) the number of sessions (4–20, 21–40, 41–60 and more than 60 sessions); 2) the number of sessions per week (1, 2, 3, 4 and 5 days per week); and 3) the duration of each session (0–30, 31–60, 61–90 and 90–120 min).

## 3 Results

### 3.1 Study selection

**FIGURE 1:**
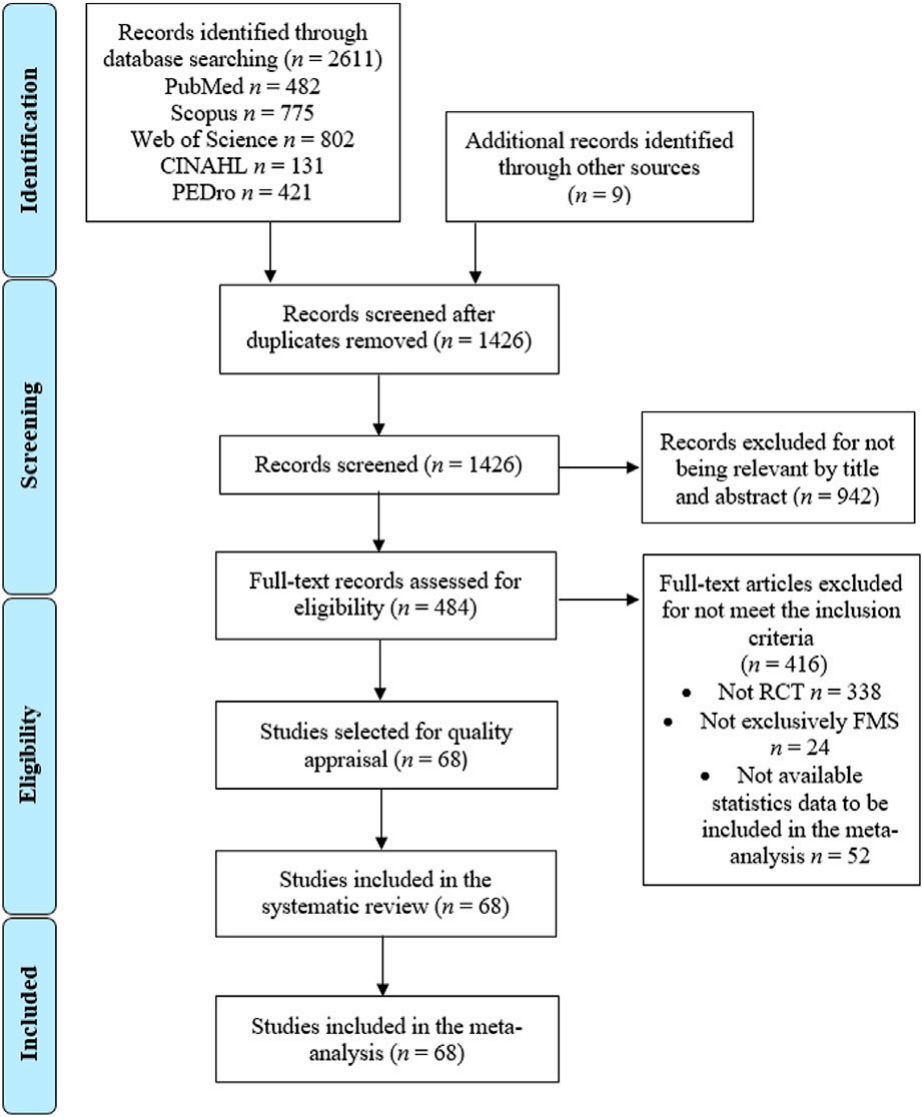
PRISMA Flow Diagram from selection of the studies.

The initial searches identified 2,620 cites and 1,426 were screened by title/abstract after to remove duplicate studies. Nine hundred forty-two were excluded for not being relevant and 416 did not meet the inclusion criteria. Ultimately, 68 studies were included in this systematic review with meta-analysis (Wigers et al., 1996; Mannerkorpi et al., 2000; Mannerkorpi et al., 2009; Gowans et al., 2001; King et al., 2002; Richards, 2002; Astin et al., 2003; Schachter et al., 2003; Cedraschi, 2004; Redondo et al., 2004; Sencan et al., 2004; Da Costa et al., 2005; Kingsley et al., 2005; Zijlstra, 2005; Gusi et al., 2006; Hammond and Freeman, 2006; Fontaine and Haaz, 2007; Munguía-Izquierdo and Legaz-Arrese, 2007; Rooks, 2007; Tomas-Carus et al., 2007; Tomas-Carus et al., 2009; Tomas-Carus et al., 2018; Tomas-Carus et al., 2021; Günendi et al., 2008; Carson et al., 2010; Fontaine et al., 2010; Sañudo Corrales et al., 2010; Arcos-Carmona et al., 2011; Núñez et al., 2011; Sañudo et al., 2011; Sañudo et al., 2015; Baptista et al., 2012; García-Martínez et al., 2012; Jones et al., 2012; Kayo et al., 2012; Castel et al., 2013; Chan et al., 2014; Chan et al., 2017; Clarke-Jenssen et al., 2014; Giannotti et al., 2014; Martín et al., 2014; Larsson et al., 2015; Latorre Román et al., 2015; Ericsson et al., 2016; Espí-López et al., 2016; Kurt, 2016; Maddali Bongi et al., 2016; Ekici et al., 2017; Windthorst et al., 2017; Assumpção et al., 2018; Kashikar-Zuck et al., 2018; Wong et al., 2018; Andrade et al., 2019; Silva et al., 2019; Atan and Karavelioğlu, 2020; Garrido-Ardila et al., 2020; Izquierdo-Alventosa et al., 2020; Izquierdo-Alventosa et al., 2020; Sauch Valmaña et al., 2020; Serrat et al., 2020; Serrat et al., 2021b; Serrat et al., 2021a; Serrat et al., 2022; Fonseca et al., 2021; Haugmark et al., 2021; Hernando-Garijo et al., 2021; Arroyo-Fernández et al., 2022; de Lorena et al., 2022). The literature searches and study selection process is shown in the PRISMA flowchart (Figure 1), which shows the number of excluded references together with the reasons.

### 3.2 Characteristics of the studies included in the review

Sixty-eight RCTs were included (Wigers et al., 1996; Mannerkorpi et al., 2000; Mannerkorpi et al., 2009; Gowans et al., 2001; King et al., 2002; Richards, 2002; Astin et al., 2003; Schachter et al., 2003; Cedraschi, 2004; Redondo et al., 2004; Sencan et al., 2004; Da Costa et al., 2005; Kingsley et al., 2005; Zijlstra, 2005; Gusi et al., 2006; Hammond and Freeman, 2006; Fontaine and Haaz, 2007; Munguía-Izquierdo and Legaz-Arrese, 2007; Rooks, 2007; Tomas-Carus et al., 2007; Tomas-Carus et al., 2009; Tomas-Carus et al., 2018; Tomas-Carus et al., 2021; Günendi et al., 2008; Carson et al., 2010; Fontaine et al., 2010; Sañudo Corrales et al., 2010; Arcos-Carmona et al., 2011; Núñez et al., 2011; Sañudo et al., 2011; Sañudo et al., 2015; Baptista et al., 2012; García-Martínez et al., 2012; Jones et al., 2012; Kayo et al., 2012; Castel et al., 2013; Chan et al., 2014; Chan et al., 2014; Clarke-Jenssen et al., 2014; Giannotti et al., 2014; Martín et al., 2014; Larsson et al., 2015; Latorre Román et al., 2015; Ericsson et al., 2016; Espí-López et al., 2016; Kurt, 2016; Maddali Bongi et al., 2016; Ekici et al., 2017; Windthorst et al., 2017; Assumpção et al., 2018; Kashikar-Zuck et al., 2018; Wong et al., 2018; Andrade et al., 2019; Silva et al., 2019; Atan and Karavelioğlu, 2020; Garrido-Ardila et al., 2020; Izquierdo-Alventosa et al., 2020; Izquierdo-Alventosa et al., 2021; Sauch Valmaña et al., 2020; Serrat et al., 2020; Serrat et al., 2021b; Serrat et al., 2021a; Serrat et al., 2022; Fonseca et al., 2021; Haugmark et al., 2021; Hernando-Garijo et al., 2021; Arroyo-Fernández et al., 2022; de Lorena et al., 2022) and reported data from 5,474 patients with FMS. The mean age was 49.23 ± 5.57 years. A total of 93% of the patients were female, and the mean body mass index (BMI) was 27.4 ± 2.39 kg/m^2^. Data on PEBT were reported from 2,893 patients with FMS (48.06 ± 5.21 years and 27.53 ± 2.38 kg/m^2^), such as circuit-based exercises or exercise-movement techniques. The duration of the proposed interventions in each study ranged from 2 to 32 weeks. The control groups consisted of 2,581 participants with FMS with a mean age of 48.91 ± 5.90 years and a mean BMI of 27.34 ± 2.67 kg/m^2^. These individuals underwent non-PEBT interventions, such as usual care, educational therapy, relaxation therapy, magnetotherapy, electrotherapy, psychology, balneotherapy or pharmacotherapy. All studies included in this review were RCTs and assessed at least one of the main outcomes (i.e., pain, the impact of FMS, QoL and anxiety). This meta-analysis includes assessments conducted just after the intervention (immediate effect) and during follow-up (12, 24 and 48 weeks). Supplementary Table S4 showed the main characteristics of the included studies.

### 3.3 Methodological quality and risk of bias

The mean methodological quality of the studies included was good (6.04 ± 1.26 points on the PEDro scale), and the risk of bias was medium. One study (1.4%) presented excellent methodological quality (Arroyo-Fernández et al., 2022), 47 studies (69.1%) presented good quality (Gowans et al., 2001; King et al., 2002; Schachter et al., 2003; Redondo et al., 2004; Da Costa et al., 2005; Kingsley et al., 2005; Hammond and Freeman, 2006; Munguía-Izquierdo and Legaz-Arrese, 2007; Rooks, 2007; Günendi et al., 2008; Mannerkorpi et al., 2009; Tomas-Carus et al., 2009; Tomas-Carus et al., 2018; Tomas-Carus et al., 2021; Carson et al., 2010; Sañudo Corrales et al., 2010; Núñez et al., 2011; Sañudo et al., 2011; Sañudo et al., 2015; Baptista et al., 2012; Jones et al., 2012; Kayo et al., 2012; Castel et al., 2013; Chan et al., 2014; 2017; Clarke-Jenssen et al., 2014; Larsson et al., 2015; Ericsson et al., 2016; Espí-López et al., 2016; Kurt, 2016; Kashikar-Zuck et al., 2018; Wong et al., 2018; Andrade et al., 2019; Silva et al., 2019; Atan and Karavelioğlu, 2020; Garrido-Ardila et al., 2020; Izquierdo-Alventosa et al., 2020; Izquierdo-Alventosa et al., 2021; Serrat et al., 2020; Serrat et al., 2021b; Serrat et al., 2021a; Serrat et al., 2022; Fonseca et al., 2021; Haugmark et al., 2021; Hernando-Garijo et al., 2021; de Lorena et al., 2022), 19 studies (27.9%) presented fair quality (Mannerkorpi et al., 2000; Richards, 2002; Astin et al., 2003; Cedraschi, 2004; Zijlstra, 2005; Gusi et al., 2006; Fontaine and Haaz, 2007; Tomas-Carus et al., 2007; Fontaine et al., 2010; Arcos-Carmona et al., 2011; García-Martínez et al., 2012; Giannotti et al., 2014; Martín et al., 2014; Latorre Román et al., 2015; Maddali Bongi et al., 2016; Ekici et al., 2017; Windthorst et al., 2017; Assumpção et al., 2018; Sauch Valmaña et al., 2020), and only 1 study (1.4%) presented poor Quality (Sencan et al., 2004). Items 5 and 6 of the PEDro scale were not met in any study, and the majority of studies showed a risk of performance bias. Assessors were not blinded in the majority of the studies, and thus, there was a risk of detection bias. Finally, some studies did not meet Item 3, indicating a possible risk of selection bias. Table 1 shows the PEDro score of each included study.

**TABLE 1:** PEDro scores for methodological quality and risk of bias assessment in the studies included in the systematic review and meta-analysis.

Study	i1	i2	i3	i4	i5	i6	i7	i8	i9	i10	i11	Total	Quality
Andrade, CP et al., 2019	Y	Y	Y	Y	N	N	Y	Y	Y	Y	Y	8	Good
Arcos Carmona, IM et al., 2011	Y	Y	N	Y	N	N	N	Y	N	Y	Y	5	Fair
Arroyo-Fernandez, R et al., 2022	Y	Y	Y	Y	Y	Y	Y	N	Y	Y	Y	9	Excellent
Assumpcao, A et al., 2018	Y	Y	N	Y	N	N	N	N	N	Y	Y	4	Fair
Astin, JA et al., 2003	Y	Y	N	Y	N	N	Y	N	N	N	Y	4	Fair
Atan and karavelioglu 2020	Y	Y	Y	Y	N	N	Y	Y	N	Y	Y	7	Good
Baptista, AS et al., 2012	Y	Y	Y	Y	N	N	Y	Y	Y	Y	Y	8	Good
Carson, JW et al., 2010	Y	Y	Y	Y	N	N	N	Y	Y	N	N	7	Good
Castel, A et al., 2013	Y	Y	N	Y	N	N	Y	Y	Y	Y	Y	7	Good
Cedraschi 2004	Y	Y	Y	Y	N	N	N	N	N	Y	Y	5	Fair
Chan, JSM et al., 2014	Y	Y	N	Y	N	N	N	Y	Y	Y	Y	6	Good
Chan, JSM et al., 2017	Y	Y	N	Y	N	N	N	Y	Y	Y	Y	6	Good
Clarke-Jenssen, A et al., 2014	Y	Y	N	Y	N	N	N	Y	Y	Y	Y	6	Good
Da Costa, D et al., 2005	Y	Y	Y	Y	N	N	Y	Y	Y	Y	Y	8	Good
Ekici, G et al., 2017	Y	Y	N	Y	N	N	Y	N	N	Y	Y	5	Fair
Ericsson, A et al., 2016	Y	Y	N	Y	N	N	Y	N	Y	Y	Y	6	Good
Espí-López, G et al., 2016	N	Y	Y	Y	N	N	Y	N	Y	Y	Y	6	Good
Fonseca et al., 2021	Y	Y	Y	Y	N	N	Y	Y	Y	Y	Y	8	Good
Fontaine and Haaz 2007	Y	Y	N	Y	N	N	N	N	N	Y	Y	4	Fair
Fontaine, KR et al., 2010	Y	Y	N	Y	N	N	N	Y	N	Y	Y	5	Fair
García-Martínez, AM et al., 2012	Y	Y	N	Y	N	N	N	N	N	Y	Y	4	Fair
Garrido-Ardila, EM et al., 2020	Y	Y	Y	Y	N	N	Y	N	N	Y	Y	6	Good
Giannotti, E et al., 2014	Y	Y	N	Y	N	N	N	Y	N	Y	Y	5	Fair
Gowans, SE et al., 2001	Y	Y	N	Y	N	N	Y	Y	Y	Y	Y	7	Good
Gunendi, Z et al., 2008	Y	Y	Y	Y	N	N	N	N	N	Y	Y	5	Good
Gusi, N et al., 2006	Y	Y	N	Y	N	N	N	Y	N	Y	Y	5	Fair
Hammond and Freeman 2006	Y	Y	Y	Y	N	N	N	N	Y	Y	Y	6	Good
Haugmark, T et al., 2021	Y	Y	Y	Y	N	N	Y	Y	Y	Y	Y	8	Good
Hernando-Garijo, I et al., 2021	Y	Y	N	Y	N	N	Y	Y	Y	Y	Y	7	Good
Izquierdo-Alventosa, R et al., 2020	Y	Y	Y	Y	N	N	Y	Y	Y	Y	Y	8	Good
Izquierdo-Alventosa, R et al., 2021	Y	Y	Y	Y	N	N	Y	Y	N	Y	Y	7	Good
Jones, KD et al., 2012	Y	Y	N	Y	N	N	N	Y	Y	Y	Y	6	Good
Kashikar-Zuck, S et al., 2018	Y	Y	Y	Y	N	N	N	Y	Y	Y	Y	7	Good
Kayo, AH et al., 2012	Y	Y	Y	Y	N	N	N	N	Y	Y	Y	6	Good
King, SJ et al., 2002	Y	Y	N	Y	N	N	Y	N	Y	Y	Y	6	Good
Kingsley, JD et al., 2005	Y	Y	N	Y	N	N	Y	N	Y	Y	Y	6	Good
Kurt 2016	Y	Y	N	Y	N	N	Y	Y	N	Y	Y	6	Good
Larsson, A et al., 2015	Y	Y	Y	Y	N	N	Y	N	Y	Y	Y	8	Good
Latorre-Román, PA et al., 2015	Y	Y	N	Y	N	N	N	Y	N	Y	Y	5	Fair
Lorena, SB et al., 2022	Y	Y	Y	Y	N	N	Y	N	N	Y	Y	6	Good
Maddali-Bongi, S et al., 2016	N	Y	N	Y	N	N	N	N	N	Y	Y	4	Fair
Mannerkorpi, K et al., 2000	Y	Y	N	Y	N	N	Y	N	N	Y	Y	5	Fair
Mannerkorpi, K et al., 2009	Y	Y	Y	Y	N	N	Y	N	Y	Y	Y	7	Good
Martín, J et al., 2014	Y	Y	N	Y	N	N	N	N	N	Y	Y	4	Fair
Munguía-Izquierdo and Legaz 2007	Y	Y	N	Y	N	N	N	Y	Y	Y	Y	6	Good
Núñez, M et al., 2011	Y	Y	Y	Y	N	N	N	Y	Y	Y	Y	7	Good
Redondo, JR et al., 2004	Y	Y	N	Y	N	N	Y	N	Y	Y	Y	6	Good
Richards 2002	Y	Y	N	N	N	N	Y	N	Y	Y	Y	5	Fair
Rooks 2007	Y	Y	Y	Y	N	N	Y	N	Y	Y	Y	7	Good
Sañudo-Corrales, B et al., 2010	Y	Y	N	Y	N	N	N	Y	Y	Y	Y	6	Good
Sañudo-Corrales et al., 2011	Y	Y	Y	Y	N	N	Y	Y	Y	Y	Y	8	Good
Sañudo-Corrales, B et al., 2015	Y	Y	Y	Y	N	N	N	Y	Y	Y	Y	7	Good
Sauch-Valmaña, G et al., 2020	N	Y	N	Y	N	N	Y	Y	N	Y	Y	5	Fair
Schachter, CL et al., 2003	Y	Y	Y	N	N	N	N	Y	Y	Y	Y	6	Good
Sencan, S et al., 2004	N	Y	N	Y	N	N	N	Y	N	Y	N	3	Poor
Serrat, M et al., 2020	Y	Y	N	Y	N	N	N	Y	Y	Y	Y	6	Good
Serrat, M et al., 2021a	Y	Y	N	Y	N	N	N	Y	Y	Y	Y	6	Good
Serrat, M et al., 2021b	Y	Y	Y	Y	N	N	Y	N	Y	Y	Y	7	Good
Serrat, M et al., 2022	Y	Y	N	Y	N	N	Y	N	Y	Y	Y	6	Good
Silva et al., 2019	Y	Y	Y	Y	N	N	Y	Y	Y	Y	Y	8	Good
Tomas-Carus, P et al., 2007	Y	Y	N	Y	N	N	N	Y	N	Y	Y	5	Fair
Tomas-Carus, P et al., 2009	Y	Y	N	Y	N	N	Y	Y	N	Y	Y	6	Good
Tomas-Carus, P et al., 2018	Y	Y	Y	Y	N	N	Y	Y	N	Y	Y	7	Good
Tomas-Carus, P et al., 2021	Y	Y	Y	Y	N	N	Y	N	N	Y	Y	6	Good
Wigers, SH et al., 1996	Y	Y	N	Y	N	N	Y	N	Y	Y	Y	6	Good
Windthorst, P et al., 2017	Y	Y	N	Y	N	N	N	N	N	Y	Y	4	Fair
Wong, A et al., 2018	Y	Y	Y	Y	N	N	N	N	N	Y	Y	5	Good
Zijlstra 2005	Y	Y	Y	Y	N	N	N	N	N	Y	Y	5	Fair

**Abbreviations:** i1, Eligibility criteria; i2, Random allocation; i3, Concealed allocation; i4, Baseline comparability; i5, Blind subjects; i6, Blind therapists; i7, Blind assessors; i8, Adequate follow-up; i9, Intention-to-treat analysis; i10, Between-group comparisons; i11, Point estimates and variability; Y, yes; N, No. Note: Eligibility criteria item does not contribute to total score.

### 3.4 Synthesis of variables

The studies included provided data for each variable thorough the following questionnaires or measures. Pain was assessed with data from the VAS, the NPRS, the Brief Pain Inventory (BPI), the FIQ-pain dimension and the Pain Catastrophizing Scale (PCS). The impact of FMS was assessed with data from the FIQ. QoL was assessed using data from the SF-36. Anxiety was analyzed using data from the BAI, the State Trait Anxiety Inventory (STAI), the Psychological General Wellbeing (PGWB), the HADS-anxiety dimension, the Arthritis Impact Measurement Scales (AIMS), the VAS for anxiety, and the FIQ-anxiety dimension.

### 3.5 Effects of PEBT on pain

**FIGURE 2:**
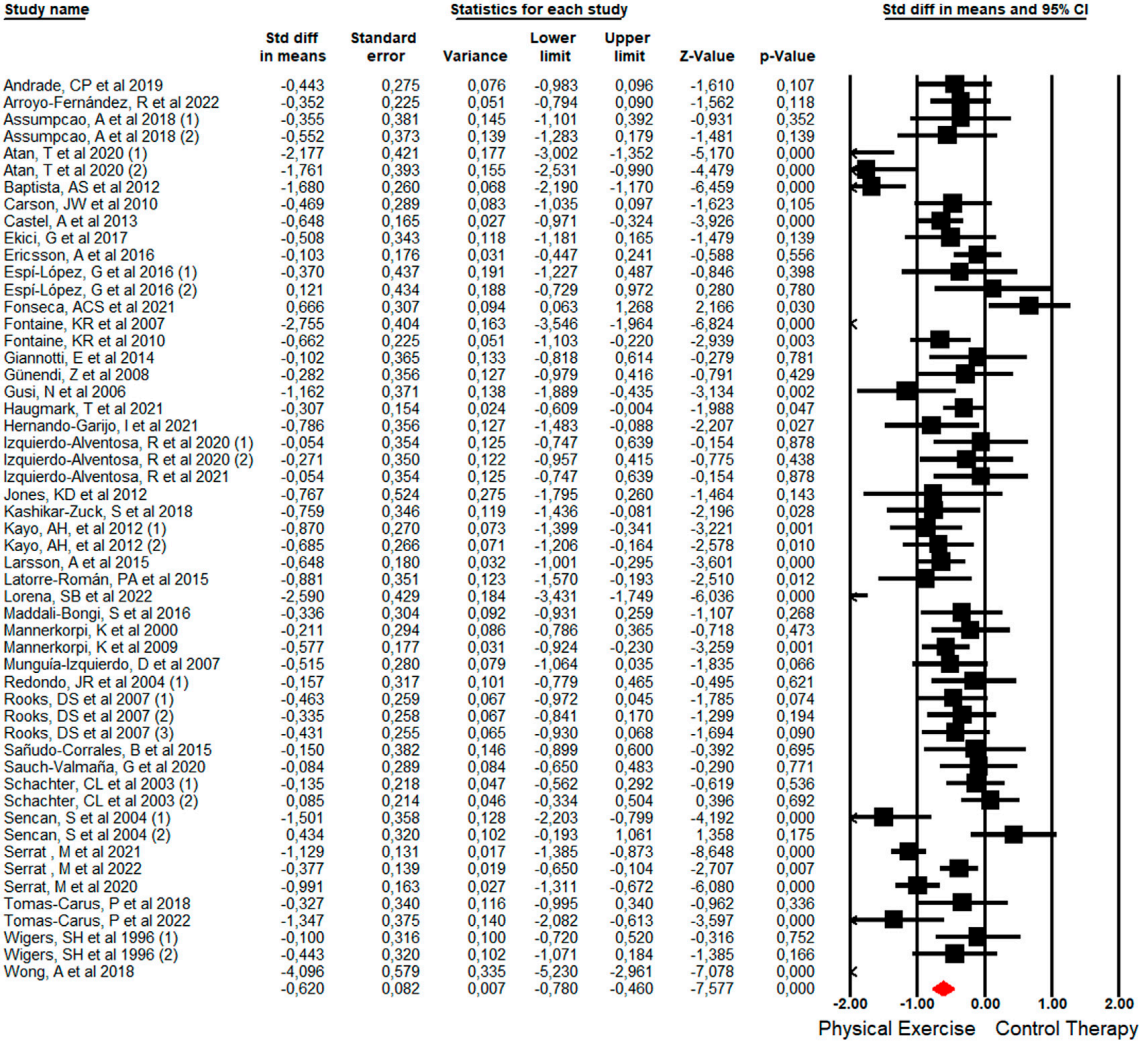
Forest Plot of the immediate effect of PEBT on pain.

Forty-seven studies (Wigers et al., 1996; Mannerkorpi et al., 2000; Mannerkorpi et al., 2009; Schachter et al., 2003; Cedraschi, 2004; Redondo et al., 2004; Sencan et al., 2004; Zijlstra, 2005; Gusi et al., 2006; Hammond and Freeman, 2006; Fontaine and Haaz, 2007; Munguía-Izquierdo and Legaz-Arrese, 2007; Rooks, 2007; Günendi et al., 2008; Carson et al., 2010; Fontaine et al., 2010; Baptista et al., 2012; Jones et al., 2012; Kayo et al., 2012; Castel et al., 2013; Clarke-Jenssen et al., 2014; Giannotti et al., 2014; Larsson et al., 2015; Latorre Román et al., 2015; Sañudo et al., 2015; Ericsson et al., 2016; Espí-López et al., 2016; Ekici et al., 2017; Assumpção et al., 2018; Kashikar-Zuck et al., 2018; Tomas-Carus et al., 2018; Wong et al., 2018; Andrade et al., 2019; Atan and Karavelioğlu, 2020; Izquierdo-Alventosa et al., 2020; Izquierdo-Alventosa et al., 2021; Sauch Valmaña et al., 2020; Serrat et al., 2021b; Serrat et al., 2022; Fonseca et al., 2021; Haugmark et al., 2021; Hernando-Garijo et al., 2021; Arroyo-Fernández et al., 2022; de Lorena et al., 2022) provided data regarding the effect of PEBT on reducing pain in comparison to other interventions. The majority of these studies (*n* = 43, with 53 independent comparisons) assessed the immediate effect of PEBT on pain, with a moderate quality of evidence. There was medium-sized effect indicating the superiority of PEBT for reducing pain (SMD = −0.62; 95% CI −0.78 to −0.46; *p* < 0.001) (Table 2; Figure 2). In addition, PEBT led to a 1.4-point reduction in the VAS for pain (95% CI −1.5–−1.27; *p* < 0.001). Visual analysis of the forest plot revealed asymmetry, thus indicating a high risk of publication bias (Egger *p* = .16). The trim-and-fill estimation method revealed 32% variation from the original effect (SMD = −0.8) ((Supplementary Figure S1). This indicates that the original pooled effect is underestimated due to the risk of publication bias. The level of heterogeneity was low-to-moderate (I^2^ = 34.1%; Q = 78.9 (df = 52); *p* = 0.01). Sensitivity analysis showed a maximum variation of 2.1% from the original pooled effect.

**TABLE 2:** Main findings of the meta-analysis to assess the immediate effect of PEBT.

Variables	Effect size				Heterogeneity			Publication bias			GRADE framework					
	K	SMD	95% CI	P	Q (df)	I^2^ (%)	P	Funnel plot P)	Trim-and-fill		Risk of bias	Incons	Indir	Imprec	Pub bias	Quality
									Adj SMD	% Var						
Pain	53	−0.62	−0.51 to −0.46	<0.001	78.9 (52)	34.1	0.01	Asym. (0.16)	−0.80	32	Med	Yes	No	No	Yes	Moderate
FMS Impact	55	−0.52	−0.67 to −0.36	<0.001	90.4 (54)	40.3	0.01	Asym. (0.52)	−0.71	29	Med	Yes	No	No	Yes	Moderate
QoL-Physical	27	0.51	0.33 to 0.69	<0.001	22.4 (26)	8.7	0.66	Sym. (0.48)	0.56	9	Med	No	No	No	No	High
QoL-Mental	23	0.48	0.29 to 0.67	<0.001	25.6 (22)	14.1	0.27	Asym. (0.01)	0.48	0	Med	No	No	No	No	High
Anxiety	30	−0.36	−0.49 to −0.25	<0.001	31.14 (29)	7.68	0.37	Sym. (0.66)	−0.36	0	Med	No	No	No	No	High

**Abbreviations:** K, number of comparisons; SMD, standardized mean difference; 95% CI, 95% Confidence interval; p, *p*-value; Q, Q-test; df, degree of freedom; I2, degree of inconsistency; Adj, Adjusted; % var, Percentage of variation; Incons, Inconsistency; Indir, Indirect evidence; Imprec, Imprecision; Pub bias; Publication bias; Asym, Asymmetric; Sym, Symmetric; Med, Medium; QoL, quality of life.

To examine the effects of specific modes of PEBT on pain reduction, we performed subgroup analysis. Circuit-based exercises had a medium-sized effect on pain reduction (SMD = −0.54; 95% CI −0.72 to −0.38; *p* < 0.001), and exercise-movement technique had a large-sized effect on pain reduction (SMD = −1.1; 95% CI −1.48–−0.63; *p* < 0.001); the quality of evidence was high ((Supplementary Table S2).

Additional subgroup analyses were performed to assess the effect of PEBT on pain over time. Our results showed that PEBT had a medium-to large-sized effect on pain at the 12-week follow-up (SMD = −0.74; 95% CI −1.03–−0.45; *p* < 0.001) and a small-sized effect at the 24-week follow-up (SMD = −0.19; 95% CI −0.32–−0.06; *p* = 0.04). No effect was found at 48 weeks (SMD = −0.04; 95% CI −0.32–0.24; *p* = 0.78) (Supplementary Table S2).

Our findings revealed that the most effective dose of PEBT for reducing pain in patients with FMS was 21–40 sessions (SMD = −0.83; 95% CI −1.1 to −0.56; *p* < 0.001), 3 sessions per week (SMD = −0.82; 95% CI −1.2–−0.48; *p* < 0.001), and 61–90 min per session (SMD −1.08; 95% CI −1.55–−0.62; *p* < 0.001) (Supplementary Table S3).

### 3.6 Effects of PEBT on the impact of FMS

**FIGURE 3:**
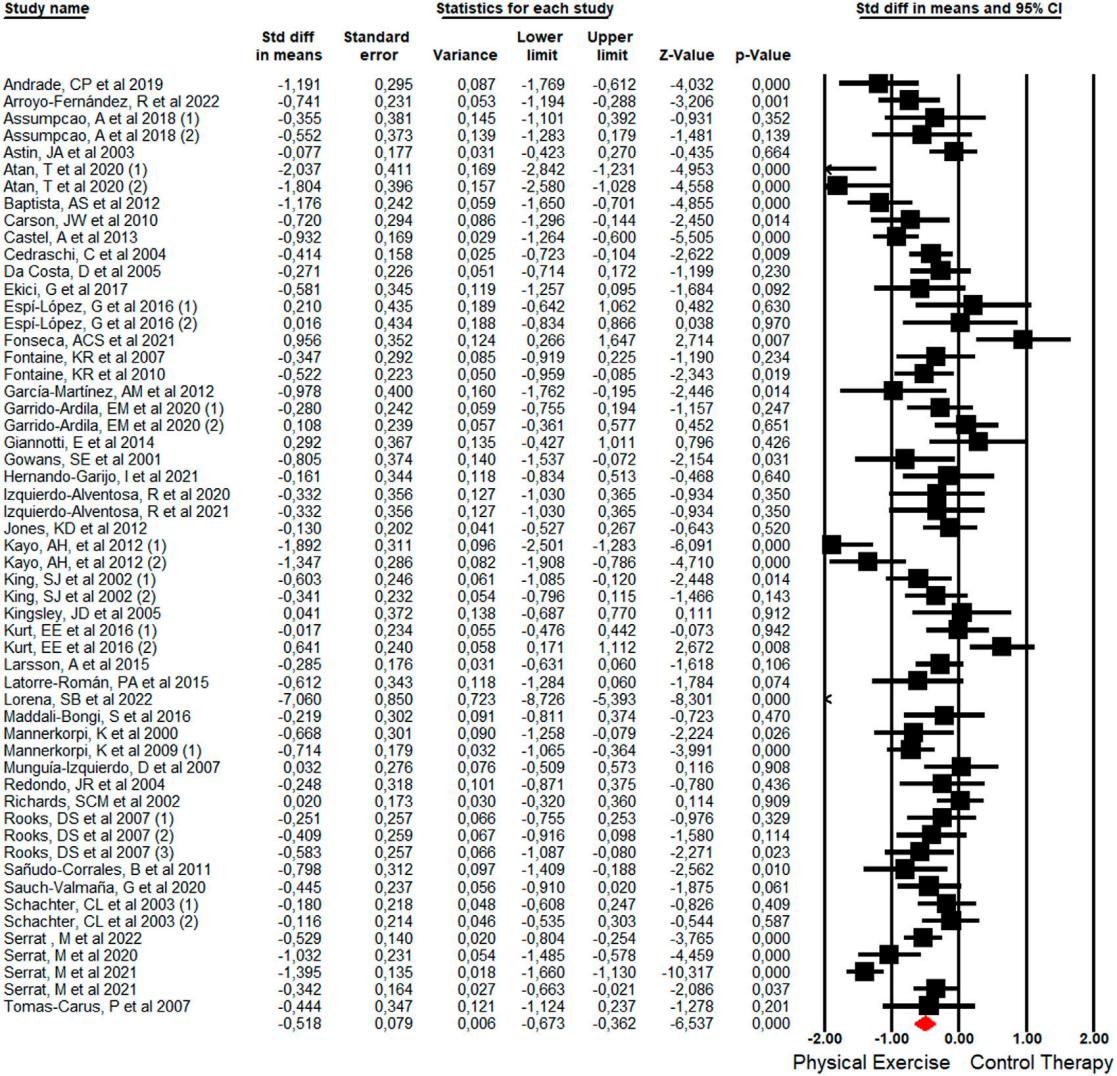
Forest Plot of the immediate effect of PEBT on FMS impact.

Forty-nine studies (Mannerkorpi et al., 2000; Mannerkorpi et al., 2009; Gowans et al., 2001; King et al., 2002; Richards, 2002; Astin et al., 2003; Schachter et al., 2003; Cedraschi, 2004; Redondo et al., 2004; Da Costa et al., 2005; Kingsley et al., 2005; Zijlstra, 2005; Hammond and Freeman, 2006; Fontaine and Haaz, 2007; Munguía-Izquierdo and Legaz-Arrese, 2007; Rooks, 2007; Tomas-Carus et al., 2007; 2018; Carson et al., 2010; Fontaine et al., 2010; Sañudo et al., 2011; Baptista et al., 2012; García-Martínez et al., 2012; Jones et al., 2012; Kayo et al., 2012; Castel et al., 2013; Giannotti et al., 2014; Martín et al., 2014; Larsson et al., 2015; Latorre Román et al., 2015; Espí-López et al., 2016; Kurt, 2016; Maddali Bongi et al., 2016; Ekici et al., 2017; Assumpção et al., 2018; Andrade et al., 2019; Atan and Karavelioğlu, 2020; Garrido-Ardila et al., 2020; Izquierdo-Alventosa et al., 2020; Izquierdo-Alventosa et al., 2021; Sauch Valmaña et al., 2020; Serrat et al., 2020; Serrat et al., 2021b; Serrat et al., 2021a; Serrat et al., 2022; Fonseca et al., 2021; Hernando-Garijo et al., 2021; Arroyo-Fernández et al., 2022; de Lorena et al., 2022) assessed the effect of PEBT on the impact of FMS. Data from 45 studies (with 55 independent comparisons) with a moderate quality of evidence were analyzed, and there was a medium-sized effect indicating the superiority of PEBT compared to other interventions in the immediate term (SMD = −0.52; 95% CI −0.67 to −0.36; *p* < 0.001) (Table 2; Figure 3). In addition, PEBT led to a 7.5-point reduction in scores on the FIQ questionnaire (95% CI −8.14 to −5.1; *p* < 0.001). Trim-and-fill estimation revealed a variation of 29% from the original pooled effect (SMD adjusted = −0.71), thus indicating potential publication bias. Similar to the findings for pain reduction, the publication bias observed for this outcome indicates that the true effect of PEBT on FIQ was underestimated (Supplementary Figure S2). The level of heterogeneity was moderate (I^2^ = 40.3%; Q = 90.4 (df = 54); *p* = .01), and sensitivity analysis did not reveal substantial variations (5.2%) from the original effect size.

Subgroup analysis based on the specific mode of PEBT used showed a medium-sized effect of circuit-based exercises (SMD = −0.54; 95% CI −0.71 to −0.38; *p* < 0.001) and a low-to medium-sized effect of exercise movement techniques (SMD = −0.32; 95% CI −0.48 to −0.15; *p* = 0.001) on reducing the impact of FMS (Supplementary Table S2).

The effect of PEBT on the impact of FMS was maintained over time and showed a medium-sized effect at 12 weeks (SMD = −0.51; 95% CI −0.84–−0.18; *p* = 0.003), 24 weeks (SMD = −0.27; 95% CI −0.41–−0.15; *p* < 0.001) and 48 weeks (SMD = −0.3; 95% CI −0.45–−0.15; *p* < 0.001) (Supplementary Table S2).

Finally, our findings reported that the most effective dose of PEBT for reducing in the impact of FMS is 21–40 sessions (SMD = −0.63; 95% CI -0.87 to −0.35; *p* < 0.001), 3 sessions per week (SMD = −0.57, 95% CI -1.03 to −0.12, *p* = 0.013), and 31–60 min per session (SMD = −0.5, 95% CI −0.7–−0.3; *p* < 0.001) (Supplementary Table S3).

### 3.7 Effects of PEBT on the QoL-physical dimension

**FIGURE 4:**
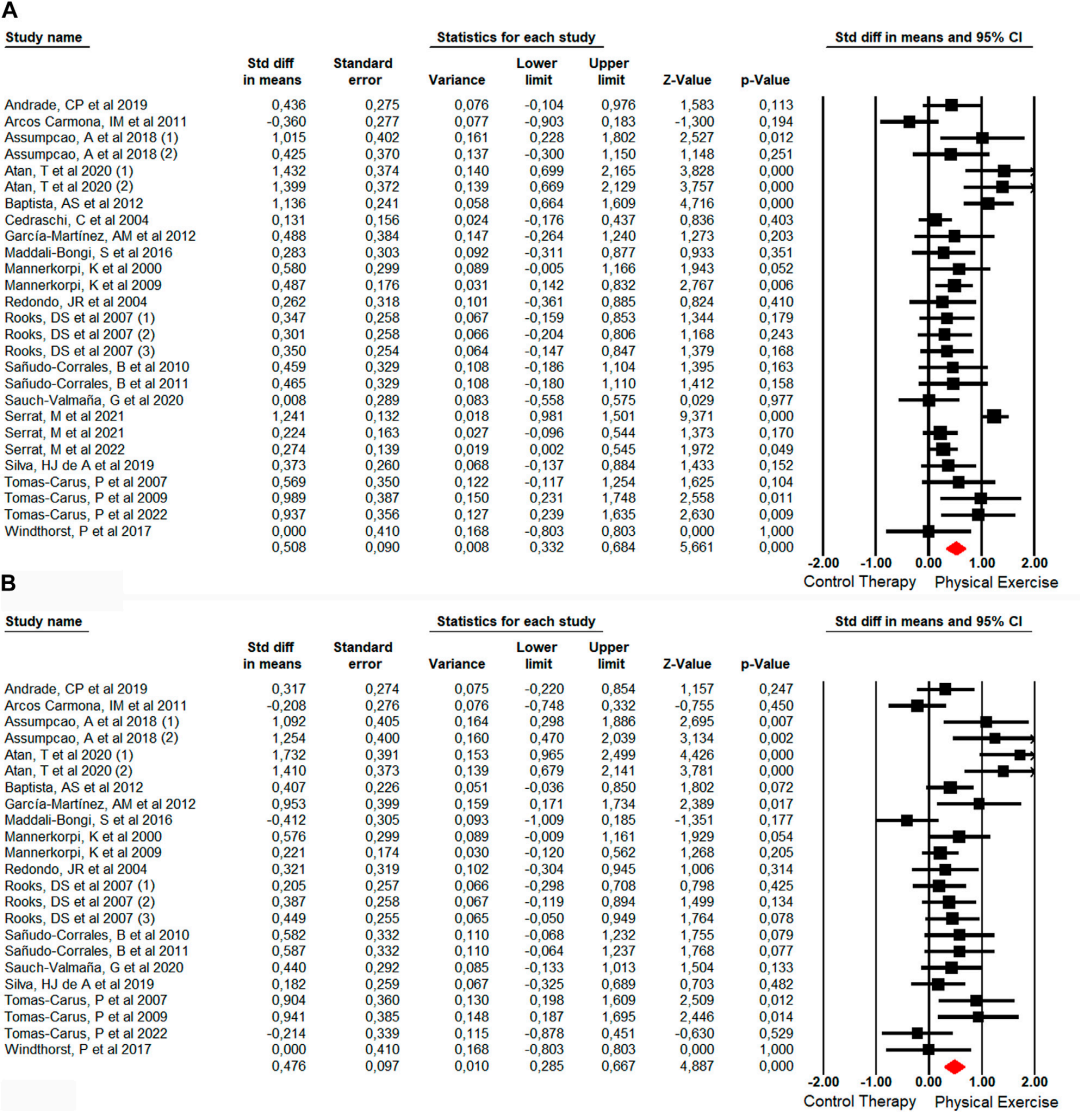
Forest Plot of the immediate effect of PEBT on QoL-physical **(A)** and mental dimension **(B)**.

Twenty-four studies (Mannerkorpi et al., 2000; Mannerkorpi et al., 2009; Cedraschi, 2004; Redondo et al., 2004; Rooks, 2007; Tomas-Carus et al., 2007; Tomas-Carus et al., 2009; Tomas-Carus et al., 2021; Sañudo Corrales et al., 2010; Arcos-Carmona et al., 2011; Núñez et al., 2011; Sañudo et al., 2011; Baptista et al., 2012; García-Martínez et al., 2012; Maddali Bongi et al., 2016; Windthorst et al., 2017; Assumpção et al., 2018; Andrade et al., 2019; Silva et al., 2019; Atan and Karavelioğlu, 2020; Sauch Valmaña et al., 2020; Serrat et al., 2021b; Serrat et al., 2021a; Serrat et al., 2022) assessed the effect of PEBT on the physical dimension of QoL. The quality of evidence was high, and there was a medium-sized effect indicating the superiority of PEBT for improving the physical dimension of QoL compared to other interventions (SMD = 0.51; 95% CI 0.33–0.69; *p* < 0.001) (Table 2; Figure 4A). Specifically, data from 23 studies with 27 independent comparisons indicated that PEBT led to a 9.7-point increase in scores on the physical component of the SF-36 when assessed immediately after therapy (95% CI 6.44–13.52; *p* < 0.001). No risk of publication bias (Egger *p* = 0.48) was found (Supplementary Figure S3), and the level of heterogeneity was very low (I^2^ = 8.7%; Q = 22.4 (df = 26); *p* = 0.66). Sensitivity analysis only showed 10.7% of variation from the original pooled effect.

Subgroup analysis based on the specific mode of PEBT used revealed a medium-sized effect of circuit-based exercises (SMD = 0.5; 95% CI 0.32–0.69; *p* < 0.001) but no effect of exercise movement techniques (SMD = 0.59; 95% CI −0.11–1.28; *p* = 0.096) (Supplementary Table S2).

Subgroup analysis based on follow-up time revealed that PEBT did not have a significant effect on physical QoL at 24 weeks (SMD = 0.21; 95% CI −0.16–0.58; *p* = .26) or 48 weeks (SMD 0.07; 95% CI −0.25–0.39; *p* = .67) (Supplementary Table S2).

The most effective dose of PEBT for increasing physical QoL is 21–40 sessions (SMD = 0.57; 95% CI 0.32–0.79; *p* < 0.001), 3 sessions per week (SMD = 0.75; 95% CI 0.24–1.24; *p =* 0.004), and 31–60 min per session (SMD 0.55; 95% CI 0.37–0.74; *p* < 0.001) (Supplementary Table S3).

### 3.8 Effects of PEBT on the QoL-mental dimension

The effect of PEBT on the mental dimension of QoL was assessed in 20 studies (Mannerkorpi et al., 2000; Mannerkorpi et al., 2009; Redondo et al., 2004; Rooks, 2007; Tomas-Carus et al., 2007; Tomas-Carus et al., 2009; Tomas-Carus et al., 2021; Sañudo Corrales et al., 2010; Arcos-Carmona et al., 2011; Núñez et al., 2011; Sañudo et al., 2011; Baptista et al., 2012; García-Martínez et al., 2012; Maddali Bongi et al., 2016; Windthorst et al., 2017; Assumpção et al., 2018; Andrade et al., 2019; Silva et al., 2019; Atan and Karavelioğlu, 2020; Sauch Valmaña et al., 2020). The quality of evidence was high, and there was a medium-sized effect indicating the superiority of PEBT for improving mental QoL compared to the other interventions when assessed immediately after therapy (SMD = 0.48; 95% CI 0.29–0.67; *p* < .001) (Table 2; Figure 4B). Across 19 studies with 23 independent comparisons, PEBT led to a 10.43-point increase in scores on the mental component of the SF-36 at immediate assessment (95% CI 6.26–14.6; *p* < 0.001). No risk of publication bias was found (Supplementary Figure S4), and the level of heterogeneity was low (I^2^ = 14.1%; Q = 20.97 (df = 22); *p* = 0.37). Sensitivity analysis showed a 6.7% variation from the original pooled effect.

Regarding the specific mode of PEBT used, there was a medium-sized effect indicating the superiority of circuit-based exercises for increasing the mental dimension of QoL (SMD = 0.54; 95% CI 0.36–0.72; *p* < 0.001) (Supplementary Table S2).

No statistically significant differences were found at 24 weeks (SMD = 0.23; 95% CI -0.13 to 0.6; *p* = 0.21) or 48 weeks (SMD = −0.07; 95% CI −0.32–0.3; *p* = 0.96) (Supplementary Table S2).

PEBT was found to be most effective at increasing scores on the mental dimension of the SF-36 when it is applied for 21–40 sessions (SMD = 0.51; 95% CI 0.28–0.73; *p* < 0.001), 5 sessions per week (SMD = 1.1; 95% CI 0.55–1.63; *p* < 0.001) and 31–60 min per session (SMD = 0.51; 95% CI 0.31– 0.71; *p* < 0.001) (Supplementary Table S3).

### 3.9 Effects of PEBT on anxiety

**FIGURE 5:**
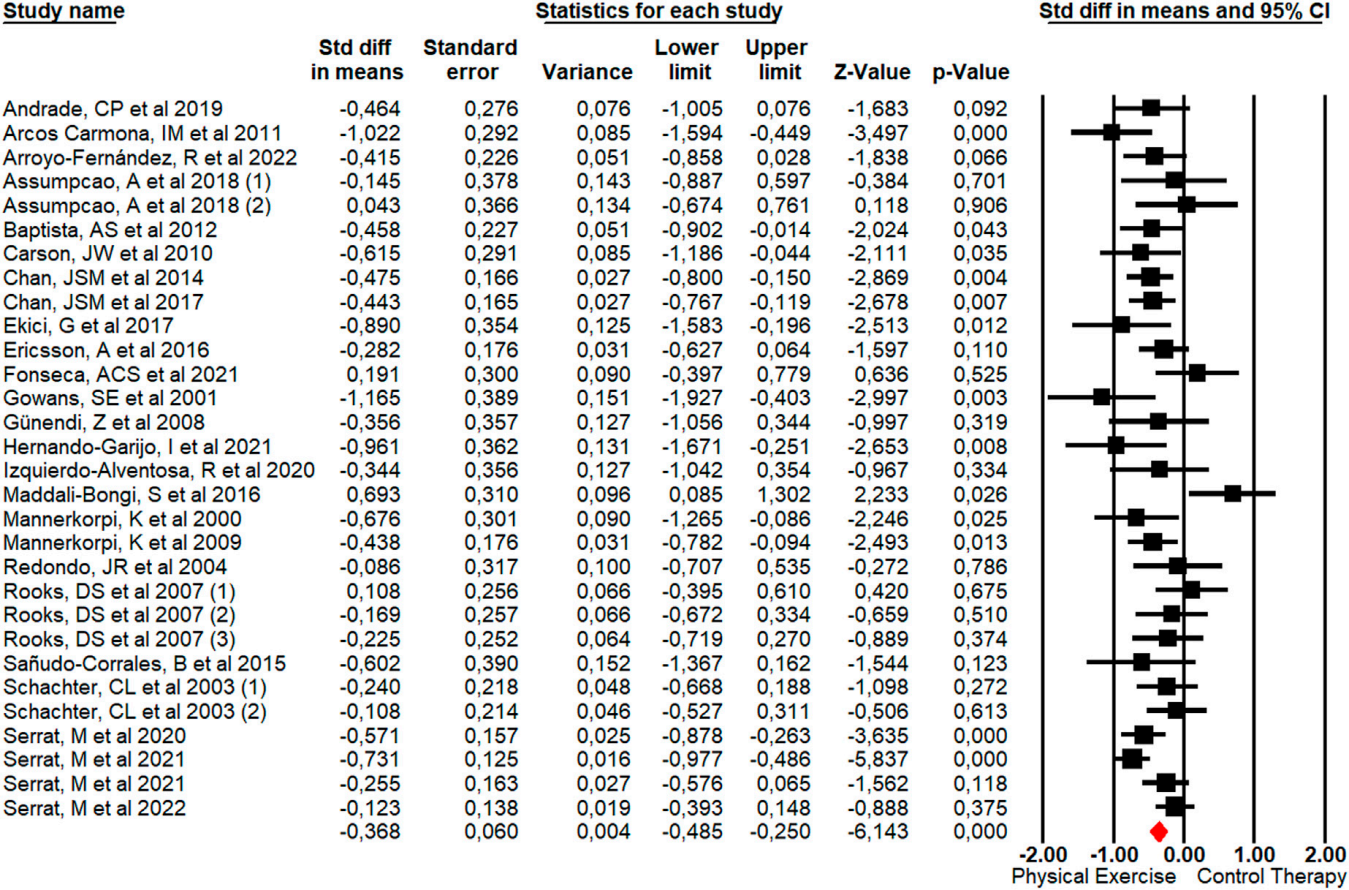
Forest Plot of the immediate effect of PEBT on anxiety.

Thirty studies (Mannerkorpi et al., 2000; Mannerkorpi et al., 2009; Gowans et al., 2001; Schachter et al., 2003; Cedraschi, 2004; Redondo et al., 2004; Zijlstra, 2005; Hammond and Freeman, 2006; Rooks, 2007; Günendi et al., 2008; Carson et al., 2010; Arcos-Carmona et al., 2011; Baptista et al., 2012; Chan et al., 2014; Chan et al., 2017; Martín et al., 2014; Sañudo et al., 2015; Ericsson et al., 2016; Maddali Bongi et al., 2016; Ekici et al., 2017; Assumpção et al., 2018; Tomas-Carus et al., 2018; Andrade et al., 2019; Izquierdo-Alventosa et al., 2020; Serrat et al., 2020; Serrat et al., 2021b; Serrat et al., 2021a; Serrat et al., 2022; Fonseca et al., 2021; Hernando-Garijo et al., 2021; Arroyo-Fernández et al., 2022) examined the effect of PEBT on anxiety. Of these, 27 studies with 30 independent comparisons assessed the immediate effect of PEBT on anxiety. The quality of evidence was high, and PEBT had a medium-sized effect on reducing anxiety compared to other interventions (SMD = −0.36; 95% CI −0.49–−0.25; *p* < 0.001) (Table 2; Figure 5). No risk of publication bias was found (Supplementary Figure S5), and the level of heterogeneity was very low (I^2^ = 7.68%; Q = 31.14 (df = 29); *p* = 0.37). Sensitivity analysis only showed a maximum variation of 9% from the original pooled effect.

Regarding the specific modes of PEBT, circuit-based exercises (SMD = −0.37; 95% CI −0.5–−0.24; *p* < 0.001) and exercise movement techniques (SMD = −0.37; 95% CI −0.66–−0.08; *p* = .013) both led to reduced levels of anxiety. Finally, PEBT showed an effect at the 12-week follow-up (SMD = −0.24; 95% CI −0.41–−0.07; *p* = 0.007) (Supplementary Table S2).

The most effective dose of PEBT for reducing anxiety in FMS patients was fewer than 20 sessions (SMD = −0.45; 95% CI −0.62–−0.3; *p* < 0.001), 3 sessions per week (SMD = −0.73; 95% CI −1.16–−0.3; *p* < 0.001) and 31–60 min per session (SMD = −0.4; 95% CI −0.51–−0.3; *p* < .001) (Supplementary Table S3).

## 4 Discussion

The present systematic review with meta-analysis included 68 RCTs that examined different modalities of PEBT, such as circuit-based exercises and movement exercise techniques. In our study, subgroup analysis was performed to determine the optimal total number of sessions, sessions per week and duration of each session to obtain optimal improvement for each assessed outcome. For all outcomes, the most effective dose of PEBT was a total of 21–40 sessions (except for anxiety, which required fewer sessions), 3 sessions per week (except for mental QoL, for which 5 sessions was optimal, even though the SMD of 3 sessions presented a higher quality of evidence due to a higher number of included studies) and between 31–60 min per session (except in pain, for which the most effective duration was 61–90 min).

To date, some reviews have assessed the effect of PEBT on different FMS symptoms. Our review differs from previous published works in the following ways: 1) it included a large number of studies; 2) it analyzed whether findings are robust by performing sensitivity and subgroup analysis; 3) it included an assessment of the quality of the studies included for each outcome and the quality of evidence for each outcome; and 4) it examined the most effective dose of PEBT for each outcome.

Additionally, our results showed that PEBT–especially circuit-based exercise (e.g., aerobic, resistance or strength exercises)—is effective at reducing pain. These findings are consistent with previous reviews, although the level of confidence in our results may be higher due to the higher level of precision in our study (Sosa-Reina et al., 2017). Furthermore, the positive effect of PEBT on pain reduction may be observed in the use of exergames (Cortés-Pérez et al., 2021) or pain neuroeducation programs (Saracoglu et al., 2022) to obtain more improvements. However, our findings did not show statistically significant differences between exercises (such as yoga or tai chi) and other therapies, in contrast to previous reviews (Cheng et al., 2019). PEBT was shown to lead to a 1.4-point reduction in scores on the pain rating scale; while this reduction does not exceed the MCID for this outcome [i.e., 2 points (Mease et al., 2011)], PEBT reduces this disabling symptom by more than 10%. Muscle gain reduces pain and is one of the most effective and fastest methods of pain control in patients with FMS (Gavi et al., 2014). Therefore, PEBT-induced improvements in muscle strength and reductions in muscle fatigue (Estévez-López et al., 2021) could explain the results obtained, i.e., the association between circuit-based exercises and pain reduction. In addition, PEBT could favor the production of endogenous opioids and beta-endorphins, causing hypoalgesia due to activation in descending nociceptive inhibitory mechanisms that decrease pain sensitivity (Tan et al., 2022).

One of the most important limitations experienced by patients with FMS occurs as a consequence of the enormous impact on all areas of their lives, including physical, psychological and work factors, as assessed by the FIQ (Bennett et al., 2007). Our findings suggest, with high-quality evidence, that PEBT is effective in reducing the impact of FMS; PEBT led to a 7.5-point reduction in the FIQ total score, which is less than the MCID [14% (BENNETT et al., 2009)]. Consistent with previous reviews (Kim et al., 2019; Galvão-Moreira et al., 2021), our findings showed that circuit-based exercise (including in water-based exercises) and movement-exercise techniques are effective in reducing the impact of FMS, with circuit-based exercises being the most effective mode of PEBT. This effect may be related to the fact that circuit-based exercises are the best option for reducing FMS-related pain. The reduction in pain reduces kinesiophobia and increases the level of activity of these patients (Martinez-Calderon et al., 2021), thereby limiting the negative impact of FMS symptoms.

Regarding psychological dimensions, PEBT leads to a reduction in anxiety; however, in contrast to other outcomes, exercise-movement technique is the most effective mode of PEBT for this outcome. Tai Chi, yoga and meditation exercises as monotherapy or adjunctive therapy have been shown to reduce anxiety, depression or sleep disorders (Saeed et al., 2019). For example, yoga has shown interesting results in reducing pain, anxiety and catastrophizing among individuals with FMS, thereby increasing their functional capacity and QoL (Lazaridou et al., 2019). PEBT led to a 9-7-point increase and a 10.43-point increase in physical and mental SF-36 scores, respectively. Thus, PEBT is considered an excellent therapy for improving QoL because these values are important clinical differences between pre- and posttreatment. Circuit-based exercises are the most effective mode of PEBT for improving QoL. However, the very low number of studies examining the effects of exercise-movement techniques may mask any potential effect. Finally, although exercise is effective at reducing anxiety, the effect of both exercise modalities was similar. However, the number of studies that reported data from circuit-based exercise was notably large.

Our findings suggest that the most effective dose of PEBT for pain management is three sessions per week for 2–4 months (21–40 sessions) with each session lasting 60–90 min. Sessions lasting 30–60 min are recommended for managing the impact of the disease. In contrast, Sosa-Reina proposed a shorter duration of sessions (30–60 min), a longer total period of therapy (4–6 months) and the same weekly frequency to observe the strongest effects on FMS management (Sosa-Reina et al., 2017). In addition, the results obtained in this review showed that a duration of 3–6 months is necessary to observe improvements in depressive symptoms (Sosa-Reina et al., 2017). However, according to our results, a reduction in anxiety levels could be observed between 1 week and 2 months if the exercise is performed at least 3 days per week with a duration of 30–60 min.

Although our findings have several clinical implications, some limitations must be considered. First, one of the challenges in achieving the objectives of this study was the considerable diversity and variability of PEBT modes examined across studies; even though we categorized these therapies into two groups, there was still heterogeneity within each group. The risk of publication bias for some outcomes indicates that the original pooled effect of PEBT on pain and the impact of FMS was underestimated. For these outcomes, the quality of evidence was downgraded due to the trim and fill estimation method yielding variations of greater than 10%. In addition, it is important to take into account the possible risk of selection, detection and performance biases, which could decrease the quality of evidence of our findings. Another limitation is the large variability in the control groups, as there was a small number of studies for each type of control therapy. Finally, the number of studies that reported data on circuit-based exercise was greater than the number of studies that reported data on exercise-movement technique; thus, findings regarding the latter modality must be interpreted with caution.

## 5 Conclusion

PEBT is effective in reducing pain, the impact of FMS and anxiety and increasing physical and mental QoL in patients with FMS in comparison to other classical therapeutic options, such as electrotherapy, balneotherapy, drugs, relaxation or usual care. Circuit-based exercises (e.g., aerobic, strength, flexibility, resistance exercises) are effective for all these outcomes, especially for reducing FMS impact and increasing physical and mental QoL, while exercise-movement techniques (e.g., Tai Chi, yoga, Pilates) are especially effective for reducing pain and anxiety. It is possible that exercise movement techniques may be effective for increasing QoL, but more studies are needed to confirm this effect. In general, the most appropriate dose of PEBT is 21–40 sessions, 3 times per week, with a duration of 31–60 min; however, there are exceptions for some outcomes, such as pain and mental QoL. Finally, the effect of PEBT is not maintained over time; it weakens or disappears at 24 and 48 weeks after the end of the treatment. Our findings confirmed that PEBT is an excellent therapy used by clinicians to manage the disabling symptoms of FMS, although more research is necessary to obtain more robust findings.

## Data Availability

The datasets presented in this article are not readily available because Data are available requesting to the corresponding author. Requests to access the datasets should be directed to eobrero@ujaen.es.

## References

[B1] Allsop V. L. Schmid A. A. Miller K. K. Slaven J. E. Daggy J. K. Froman A. (2022). The pain outcomes comparing yoga vs. structured exercise (poyse) trial in veterans with fibromyalgia: Study design and methods. Front. Pain Res. 3, 934689. 10.3389/fpain.2022.934689 PMC9300933 35875477

[B2] Andrade C. P. Zamunér A. R. Forti M. Tamburús N. Y. Silva E. (2019). Effects of aquatic training and detraining on women with fibromyalgia: Controlled randomized clinical trial. Eur. J. Phys. Rehabil. Med. 55, 79–88. 10.23736/S1973-9087.18.05041-4 29984564

[B3] Araya-Quintanilla F. Gutiérrez-Espinoza H. Fuentes J. Prieto-Lafrentz F. Pavez L. Cristi-Montero C. (2022). Effectiveness of multicomponent treatment in patients with fibromyalgia: Protocol for a systematic review and meta-analysis. Syst. Rev. 11, 69. 10.1186/s13643-022-01944-1 35422009 PMC9012030

[B4] Arcos-Carmona I. M. Castro-Sánchez A. M. Matarán-Peñarrocha G. A. Gutiérrez-Rubio A. B. Ramos-González E. Moreno-Lorenzo C. (2011). Effects of aerobic exercise program and relaxation techniques on anxiety, quality of sleep, depression, and quality of life in patients with fibromyalgia: A randomized controlled trial. Med. Clin. Barc. 137, 398–401. 10.1016/j.medcli.2010.09.045 21345470

[B5] Arroyo-Fernández R. Avendaño-Coy J. Velasco-Velasco R. Palomo-Carrión R. Bravo-Esteban E. Ferri-Morales A. (2022). Effectiveness of transcranial direct current stimulation combined with exercising in people with fibromyalgia: A randomized sham-controlled clinical trial. Arch. Phys. Med. Rehabil. 103, 1524–1532. 10.1016/j.apmr.2022.02.020 35331718

[B6] Assumpção A. Matsutani L. A. Yuan S. L. Santo A. S. Sauer J. Mango P. (2018). Muscle stretching exercises and resistance training in fibromyalgia: Which is better? A three-arm randomized controlled trial. Eur. J. Phys. Rehabil. Med. 54, 663–670. 10.23736/S1973-9087.17.04876-6 29185675

[B7] Astin J. A. Berman B. M. Bausell B. Lee W.-L. Hochberg M. Forys K. L. (2003). The efficacy of mindfulness meditation plus qigong movement therapy in the treatment of fibromyalgia: A randomized controlled trial. J. Rheumatol. 30, 2257–2262. Available at: http://www.ncbi.nlm.nih.gov/pubmed/14528526. 14528526

[B8] Atan T. Karavelioğlu Y. (2020). Effectiveness of high-intensity interval training vs moderate-intensity continuous training in patients with fibromyalgia: A pilot randomized controlled trial. Arch. Phys. Med. Rehabil. 101, 1865–1876. 10.1016/j.apmr.2020.05.022 32585169

[B9] Atkins D. Best D. Briss P. A. Eccles M. Falck-Ytter Y. Flottorp S. (2004). Grading quality of evidence and strength of recommendations. BMJ 328, 1490. 10.1136/bmj.328.7454.1490 15205295 PMC428525

[B10] Bair M. J. Krebs E. E. (2020). Fibromyalgia. *Ann. Intern. Med.* 172, ITC33–ITC48. 10.7326/AITC202003030 32120395

[B11] Baptista A. S. Villela A. L. Jones A. Natour J. (2012). Effectiveness of dance in patients with fibromyalgia: A randomized, single-blind, controlled study. Clin. Exp. Rheumatol. 30, 18–23. Available at: http://www.ncbi.nlm.nih.gov/pubmed/23020850. 23020850

[B12] Bennett R. M. Bushmakin A. G. Cappelleri J. C. Zlateva G. Sadosky A. B. (2009). Minimal clinically important difference in the fibromyalgia impact questionnaire. J. Rheumatol. 36, 1304–1311. 10.3899/jrheum.081090 19369473

[B13] Bennett R. M. Jones J. Turk D. C. Russell I. J. Matallana L. (2007). An internet survey of 2,596 people with fibromyalgia. BMC Musculoskelet. Disord. 8, 27. 10.1186/1471-2474-8-27 17349056 PMC1829161

[B14] Bidonde J. Busch A. Bath B. Milosavljevic S. (2014a). Exercise for adults with fibromyalgia: An umbrella systematic review with synthesis of best evidence. Curr. Rheumatol. Rev. 10, 45–79. 10.2174/1573403X10666140914155304 25229499

[B15] Bidonde J. Busch A. J. Webber S. C. Schachter C. L. Danyliw A. Overend T. J. (2014b). Aquatic exercise training for fibromyalgia. Cochrane Database Syst. Rev., CD011336. 10.1002/14651858.CD011336 25350761 PMC10638613

[B16] Bravo C. Skjaerven L. H. Espart A. Guitard Sein-Echaluce L. Catalan-Matamoros D. (2019). Basic body awareness therapy in patients suffering from fibromyalgia: A randomized clinical trial. Physiother. Theory Pract. 35, 919–929. 10.1080/09593985.2018.1467520 29723080

[B17] Carson J. W. Carson K. M. Jones K. D. Bennett R. M. Wright C. L. Mist S. D. (2010). A pilot randomized controlled trial of the Yoga of Awareness program in the management of fibromyalgia. Pain 151, 530–539. 10.1016/j.pain.2010.08.020 20946990 PMC5568071

[B18] Cashin A. G. McAuley J. H. (2020). Clinimetrics: Physiotherapy evidence database (PEDro) scale. J. Physiother. 66, 59. 10.1016/j.jphys.2019.08.005 31521549

[B19] Castel A. Fontova R. Montull S. Periñán R. Poveda M. J. Miralles I. (2013). Efficacy of a multidisciplinary fibromyalgia treatment adapted for women with low educational levels: A randomized controlled trial. Arthritis Care Res. Hob. 65, 421–431. 10.1002/acr.21818 22899402

[B20] Cedraschi C. Desmeules J. Rapiti E. Baumgartner E. Cohen P. Finckh A. (2004). Fibromyalgia: A randomised, controlled trial of a treatment programme based on self management. Ann. Rheum. Dis. 63, 290–296. 10.1136/ard.2002.004945 14962965 PMC1754921

[B21] Chan J. S. M. Ho R. T. H. Chung K. Wang C. Yao T. Ng S. (2014). Qigong exercise alleviates fatigue, anxiety, and depressive symptoms, improves sleep quality, and shortens sleep latency in persons with chronic fatigue syndrome-like illness. Evidence-Based Complement. Altern. Med. 2014, 106048. 10.1155/2014/106048 PMC4290154 25610473

[B22] Chan J. S. M. Li A. Ng S.-M. Ho R. T. H. Xu A. Yao T.-J. (2017). Adiponectin potentially contributes to the antidepressive effects of baduanjin qigong exercise in women with chronic fatigue syndrome-like illness. Cell. Transpl. 26, 493–501. 10.3727/096368916X694238 PMC5657703 27938498

[B23] Cheng C.-A. Chiu Y.-W. Wu D. Kuan Y.-C. Chen S.-N. Tam K.-W. (2019). Effectiveness of tai chi on fibromyalgia patients: A meta-analysis of randomized controlled trials. Complement. Ther. Med. 46, 1–8. 10.1016/j.ctim.2019.07.007 31519264

[B24] Clarke-Jenssen A. Mengshoel A. Strumse Y. Forseth K. (2014). Effect of a fibromyalgia rehabilitation programme in warm versus cold climate: A randomized controlled study. J. Rehabil. Med. 46, 676–683. 10.2340/16501977-1819 24788929

[B25] Cohen J. (1977). Statistical power analysis for the behavioral sciences. New York, New York: Academic Press.

[B26] Cortés-Pérez I. Zagalaz-Anula N. Del Rocío Ibancos-Losada M. Nieto-Escámez F. A. Obrero-Gaitán E. Catalina Osuna-Pérez M. (2021). Virtual reality-based therapy reduces the disabling impact of fibromyalgia syndrome in women: Systematic review with meta-analysis of randomized controlled trials. J. Pers. Med. 11, 1167. 10.3390/JPM11111167 34834518 PMC8621064

[B27] Creed F. (2020). A review of the incidence and risk factors for fibromyalgia and chronic widespread pain in population-based studies. Pain 161, 1169–1176. 10.1097/j.pain.0000000000001819 32040078

[B28] Da Costa D. Abrahamowicz M. Lowensteyn I. Bernatsky S. Dritsa M. Fitzcharles M.-A. (2005). A randomized clinical trial of an individualized home-based exercise programme for women with fibromyalgia. Rheumatology 44, 1422–1427. 10.1093/rheumatology/kei032 16030079

[B29] de Lorena S. B. Duarte A. L. B. P. Bredemeier M. Fernandes V. M. Pimentel E. A. S. Marques C. D. L. (2022). Effects of a physical self-care support program for patients with fibromyalgia: A randomized controlled trial. J. Back Musculoskelet. Rehabil. 35, 495–504. 10.3233/BMR-191820 34657869

[B30] Del-Moral-García M. Obrero-Gaitán E. Rodríguez-Almagro D. Rodríguez-Huguet M. Osuna-Pérez M. C. Lomas-Vega R. (2020). Effectiveness of active therapy-based training to improve the balance in patients with fibromyalgia: A systematic review with meta-analysis. J. Clin. Med. 9, 3771. 10.3390/jcm9113771 33266511 PMC7700277

[B31] DerSimonian R. Laird N. (1986). Meta-analysis in clinical trials. Control. Clin. Trials 7, 177–188. 10.1016/0197-2456(86)90046-2 3802833

[B32] Duval S. Tweedie R. (2000). Trim and fill: A simple funnel-plot-based method of testing and adjusting for publication bias in meta-analysis. Biometrics 56, 455–463. 10.1111/j.0006-341X.2000.00455.x 10877304

[B33] Egger M. Smith G. D. Schneider M. Minder C. (1997). Bias in meta-analysis detected by a simple, graphical test. Bmj 315, 629–634. 10.1136/bmj.315.7109.629 9310563 PMC2127453

[B34] Ekici G. Unal E. Akbayrak T. Vardar-Yagli N. Yakut Y. Karabulut E. (2017). Effects of active/passive interventions on pain, anxiety, and quality of life in women with fibromyalgia: Randomized controlled pilot trial. Women Health 57, 88–107. 10.1080/03630242.2016.1153017 26882533

[B35] Ericsson A. Palstam A. Larsson A. Löfgren M. Bileviciute-Ljungar I. Bjersing J. (2016). Resistance exercise improves physical fatigue in women with fibromyalgia: A randomized controlled trial. Arthritis Res. Ther. 18, 176. 10.1186/s13075-016-1073-3 27473164 PMC4967304

[B36] Espí-López G. V. Inglés M. Ruescas-Nicolau M.-A. Moreno-Segura N. (2016). Effect of low-impact aerobic exercise combined with music therapy on patients with fibromyalgia. A pilot study. Complement. Ther. Med. 28, 1–7. 10.1016/j.ctim.2016.07.003 27670863

[B37] Estévez-López F. Maestre-Cascales C. Russell D. Álvarez-Gallardo I. C. Rodriguez-Ayllon M. Hughes C. M. (2021). Effectiveness of exercise on fatigue and sleep quality in fibromyalgia: A systematic review and meta-analysis of randomized trials. Arch. Phys. Med. Rehabil. 102, 752–761. 10.1016/j.apmr.2020.06.019 32721388

[B38] Estrada-Marcén N. C. Casterad-Seral J. Montero-Marin J. Serrano-Ostáriz E. (2023). Can an aerobic exercise programme improve the response of the growth hormone in fibromyalgia patients? A randomised controlled trial. Int. J. Environ. Res. Public Health 20, 2261. 10.3390/ijerph20032261 36767626 PMC9915310

[B39] Faraone S. V. (2008). Interpreting estimates of treatment effects: Implications for managed care. P T 33, 700–711. 19750051 PMC2730804

[B40] Feliu-Soler A. Borràs X. Peñarrubia-María M. T. Rozadilla-Sacanell A. D’Amico F. Moss-Morris R. (2016). Cost-utility and biological underpinnings of mindfulness-based stress reduction (MBSR) versus a psychoeducational programme (FibroQoL) for fibromyalgia: A 12-month randomised controlled trial (EUDAIMON study). BMC Complement. Altern. Med. 16, 81. 10.1186/s12906-016-1068-2 26921267 PMC4769528

[B41] Fonseca A. C. S. Faria P. C. Alcântara M. A. Pinto W. D. De Carvalho L. G. Lopes F. G. (2021). Effects of aquatic physiotherapy or health education program in women with fibromyalgia: A randomized clinical trial. Physiother. Theory Pract. 37, 620–632. 10.1080/09593985.2019.1639229 31305209

[B42] Fontaine K. R. Conn L. Clauw D. J. (2010). Effects of lifestyle physical activity on perceived symptoms and physical function in adults with fibromyalgia: Results of a randomized trial. Arthritis Res. Ther. 12, R55. 10.1186/ar2967 20353551 PMC2888205

[B43] Fontaine K. R. Haaz S. (2007). Effects of lifestyle physical activity on health status, pain, and function in adults with fibromyalgia syndrome. J. Musculoskelet. Pain 15, 3–9. 10.1300/J094v15n01_02

[B44] Franco K. F. M. Miyamoto G. C. Franco Y. R. dos S. Salvador E. M. E. S. do Nascimento B. C. B. (2023). Is Pilates more effective and cost‐effective than aerobic exercise in the treatment of patients with fibromyalgia syndrome? A randomized controlled trial with economic evaluation. Eur. J. Pain 27, 54–71. 10.1002/ejp.2039 36097826

[B45] Frange C. Hirotsu C. Hachul H. Araujo P. Tufik S. Andersen M. L. (2014). Fibromyalgia and sleep in animal models: A current overview and future directions. Curr. Pain Headache Rep. 18, 434. 10.1007/s11916-014-0434-3 24908494

[B46] Galvão-Moreira L. V. de Castro L. O. Moura E. C. R. de Oliveira C. M. B. Nogueira Neto J. Gomes L. M. R. de S. (2021). Pool-based exercise for amelioration of pain in adults with fibromyalgia syndrome: A systematic review and meta-analysis. Mod. Rheumatol. 31, 904–911. 10.1080/14397595.2020.1829339 32990113

[B47] Galvez-Sánchez C. M. Duschek S. Reyes del Paso G. A. (2019). Psychological impact of fibromyalgia: Current perspectives. Psychol. Res. Behav. Manag. 12, 117–127. 10.2147/PRBM.S178240 30858740 PMC6386210

[B48] Galvez-Sánchez C. M. Reyes del Paso G. A. (2020). Diagnostic criteria for fibromyalgia: Critical review and future perspectives. J. Clin. Med. 9, 1219. 10.3390/jcm9041219 32340369 PMC7230253

[B49] García-Martínez A. M. De Paz J. A. Márquez S. (2012). Effects of an exercise programme on self-esteem, self-concept and quality of life in women with fibromyalgia: A randomized controlled trial. Rheumatol. Int. 32, 1869–1876. 10.1007/s00296-011-1892-0 21442171

[B50] Garrido-Ardila E. M. González-López-Arza M. V. Jiménez-Palomares M. García-Nogales A. Rodríguez-Mansilla J. (2020). Effectiveness of acupuncture vs. core stability training in balance and functional capacity of women with fibromyalgia: A randomized controlled trial. Clin. Rehabil. 34, 630–645. 10.1177/0269215520911992 32204612

[B51] Gavi M. B. R. O. Vassalo D. V. Amaral F. T. Macedo D. C. F. Gava P. L. Dantas E. M. (2014). Strengthening exercises improve symptoms and quality of life but do not change autonomic modulation in fibromyalgia: A randomized clinical trial. PLoS One 9, e90767. 10.1371/journal.pone.0090767 24651512 PMC3961245

[B52] Giannotti E. Koutsikos K. Pigatto M. Rampudda M. E. Doria A. Masiero S. (2014). Medium-/long-term effects of a specific exercise protocol combined with patient education on spine mobility, chronic fatigue, pain, aerobic fitness and level of disability in fibromyalgia. Biomed. Res. Int. 2014, 474029. 10.1155/2014/474029 24616894 PMC3925511

[B53] Giesecke T. Gracely R. H. Grant M. A. B. Nachemson A. Petzke F. Williams D. A. (2004). Evidence of augmented central pain processing in idiopathic chronic low back pain. Arthritis Rheum. 50, 613–623. 10.1002/art.20063 14872506

[B54] Gota C. E. (2022). Fibromyalgia: Recognition and management in the primary care office. Fibromyalgia. *Rheum. Dis. Clin. North Am.* 48, 467–478. 10.1016/j.rdc.2022.02.006 35400372

[B55] Goubert D. De Pauw R. Meeus M. Willems T. Cagnie B. Schouppe S. (2017). Lumbar muscle structure and function in chronic versus recurrent low back pain: A cross-sectional study. Spine J. 17, 1285–1296. 10.1016/j.spinee.2017.04.025 28456669

[B56] Gowans S. E. DeHueck A. Voss S. Silaj A. Abbey S. E. Reynolds W. J. (2001). Effect of a randomized, controlled trial of exercise on mood and physical function in individuals with fibromyalgia. Arthritis Rheum. 45, 519–529. 10.1002/1529-0131(200112)45:6<519:AID-ART377>3.0.CO;2-3 11762686

[B57] Günendi Z. Meray J. Özdem S. (2008). The effect of a 4-week aerobic exercise program on muscle performance in patients with fibromyalgia. J. Back Musculoskelet. Rehabil. 21, 185–191. 10.3233/BMR-2008-21306

[B58] Gusi N. Tomas-Carus P. Häkkinen A. Häkkinen K. Ortega-Alonso A. (2006). Exercise in waist-high warm water decreases pain and improves health-related quality of life and strength in the lower extremities in women with fibromyalgia. Arthritis Rheum. 55, 66–73. 10.1002/art.21718 16463415

[B59] Hammond A. Freeman K. (2006). Community patient education and exercise for people with fibromyalgia: A parallel group randomized controlled trial. Clin. Rehabil. 20, 835–846. 10.1177/0269215506072173 17008336

[B60] Haugmark T. Hagen K. B. Provan S. A. Smedslund G. Zangi H. A. (2021). Effects of a mindfulness-based and acceptance-based group programme followed by physical activity for patients with fibromyalgia: A randomised controlled trial. a Open 11, e046943. 10.1136/bmjopen-2020-046943 PMC8245472 34187823

[B61] Häuser W. Ablin J. Fitzcharles M.-A. Littlejohn G. Luciano J. V. Usui C. (2015). Fibromyalgia. Nat. Rev. Dis. Prim. 1, 15022. 10.1038/nrdp.2015.22.[Suggestion Available for atl, stl from External Pubmed] [CS: 100]. 27189527 10.1038/nrdp.2015.22

[B62] Hernando-Garijo I. Ceballos-Laita L. Mingo-Gómez M. T. Medrano-de-la-Fuente R. Estébanez-de-Miguel E. Martínez-Pérez M. N. (2021). Immediate effects of a telerehabilitation program based on aerobic exercise in women with fibromyalgia. Int. J. Environ. Res. Public Health 18, 2075. 10.3390/ijerph18042075 33672691 PMC7924356

[B63] Higgins J. Thomas J. (2020). Cochrane Handbook for systematic reviews of interventions. 2nd ed. Hoboken, NJ: Wiley Blackwell and Sons.

[B64] Higgins J. Thompson S. Deeks J. Altman D. (2003). Measuring inconsistency in meta-analyses. BMJ 327, 557–560. 10.1136/bmj.327.7414.557 12958120 PMC192859

[B65] Higgins J. Thompson S. Deeks J. Altman D. (2002). Statistical heterogeneity in systematic reviews of clinical trials: A critical appraisal of guidelines and practice. J. Heal. Serv. Res. Policy 7, 51–61. 10.1258/1355819021927674 11822262

[B66] Hozo S. P. Djulbegovic B. Hozo I. (2005). Estimating the mean and variance from the median, range, and the size of a sample. BMC Med. Res. Methodol. 5, 13. 10.1186/1471-2288-5-13 15840177 PMC1097734

[B67] Izquierdo-Alventosa R. Inglés M. Cortés-Amador S. Gimeno-Mallench L. Chirivella-Garrido J. Kropotov J. (2020). Low-intensity physical exercise improves pain catastrophizing and other psychological and physical aspects in women with fibromyalgia: A randomized controlled trial. Int. J. Environ. Res. Public Health 17, 3634. 10.3390/ijerph17103634 32455853 PMC7277480

[B68] Izquierdo-Alventosa R. Inglés M. Cortés-Amador S. Gimeno-Mallench L. Sempere-Rubio N. Serra-Añó P. (2021). Effectiveness of high-frequency transcranial magnetic stimulation and physical exercise in women with fibromyalgia: A randomized controlled trial. Phys. Ther. 101, pzab159. 10.1093/ptj/pzab159 34216139

[B69] Jones K. D. Sherman C. A. Mist S. D. Carson J. W. Bennett R. M. Li F. (2012). A randomized controlled trial of 8-form Tai chi improves symptoms and functional mobility in fibromyalgia patients. Clin. Rheumatol. 31, 1205–1214. 10.1007/s10067-012-1996-2 22581278 PMC5571653

[B70] Kashikar-Zuck S. Black W. R. Pfeiffer M. Peugh J. Williams S. E. Ting T. V. (2018). Pilot randomized trial of integrated cognitive-behavioral therapy and neuromuscular training for juvenile fibromyalgia: The FIT teens program. J. Pain 19, 1049–1062. 10.1016/j.jpain.2018.04.003 29678563 PMC6119635

[B71] Kayo A. H. Peccin M. S. Sanches C. M. Trevisani V. F. M. (2012). Effectiveness of physical activity in reducing pain in patients with fibromyalgia: A blinded randomized clinical trial. Rheumatol. Int. 32, 2285–2292. 10.1007/s00296-011-1958-z 21594719

[B72] Kim S. Y. Busch A. J. Overend T. J. Schachter C. L. van der Spuy I. Boden C. (2019). Flexibility exercise training for adults with fibromyalgia. Cochrane Database Syst. Rev. 9, CD013419. 10.1002/14651858.CD013419 31476271 PMC6718217

[B73] King S. J. Wessel J. Bhambhani Y. Sholter D. Maksymowych W. (2002). The effects of exercise and education, individually or combined, in women with fibromyalgia. J. Rheumatol. 29, 2620–2627. Available at: http://www.ncbi.nlm.nih.gov/pubmed/12465163. 12465163

[B74] Kingsley J. D. Panton L. B. Toole T. Sirithienthad P. Mathis R. McMillan V. (2005). The effects of a 12-week strength-training program on strength and functionality in women with fibromyalgia. Arch. Phys. Med. Rehabil. 86, 1713–1721. 10.1016/j.apmr.2005.04.014 16181932

[B75] Kurt E. E. Kocak F. A. Erdem H. R. Tuncay F. Kelez F. (2016). Which non-pharmacological treatment is more effective on clinical parameters in patients with fibromyalgia: Balneotherapy or aerobic exercise? Arch. Rheumatol. 31, 162–169. 10.5606/ArchRheumatol.2016.5751 29900959 PMC5827833

[B76] Larsson A. Palstam A. Löfgren M. Ernberg M. Bjersing J. Bileviciute-Ljungar I. (2015). Resistance exercise improves muscle strength, health status and pain intensity in fibromyalgia—A randomized controlled trial. Arthritis Res. Ther. 17, 161. 10.1186/s13075-015-0679-1 26084281 PMC4489359

[B77] Latorre Román P. Á. Santos e Campos M. A. García-Pinillos F. (2015). Effects of functional training on pain, leg strength, and balance in women with fibromyalgia. Mod. Rheumatol. 25, 943–947. 10.3109/14397595.2015.1040614 25867230

[B78] Lazaridou A. Koulouris A. Devine J. K. Haack M. Jamison R. N. Edwards R. R. (2019). Impact of daily yoga-based exercise on pain, catastrophizing, and sleep amongst individuals with fibromyalgia. J. Pain Res. 12, 2915–2923. 10.2147/JPR.S210653 31802932 PMC6802732

[B79] Macedo L. G. Elkins M. R. Maher C. G. Moseley A. M. Herbert R. D. Sherrington C. (2010). There was evidence of convergent and construct validity of Physiotherapy Evidence Database quality scale for physiotherapy trials. J. Clin. Epidemiol. 63, 920–925. 10.1016/j.jclinepi.2009.10.005 20171839

[B80] Macfarlane G. J. Kronisch C. Dean L. E. Atzeni F. Häuser W. Fluß E. (2017). EULAR revised recommendations for the management of fibromyalgia. Ann. Rheum. Dis. 76, 318–328. 10.1136/annrheumdis-2016-209724 27377815

[B81] Maddali Bongi S. Paoletti G. Calà M. Del Rosso A. El Aoufy K. Mikhaylova S. (2016). Efficacy of rehabilitation with tai ji quan in an Italian cohort of patients with fibromyalgia syndrome. Complement. Ther. Clin. Pract. 24, 109–115. 10.1016/j.ctcp.2016.05.010 27502810

[B82] Mannerkorpi K. Nordeman L. Ericsson A. Arndorw M. GAU Study Group (2009). Pool exercise for patients with fibromyalgia or chronic widespread pain: A randomized controlled trial and subgroup analyses. J. Rehabil. Med. 41, 751–760. 10.2340/16501977-0409 19774310

[B83] Mannerkorpi K. Nyberg B. Ahlmén M. Ekdahl C. (2000). Pool exercise combined with an education program for patients with fibromyalgia syndrome. A prospective, randomized study. J. Rheumatol. 27, 2473–2481. Available at: http://www.ncbi.nlm.nih.gov/pubmed/11036846. 11036846

[B84] Martín J. Torre F. Padierna A. Aguirre U. González N. Matellanes B. (2014). Impact of interdisciplinary treatment on physical and psychosocial parameters in patients with fibromyalgia: Results of a randomised trial. Int. J. Clin. Pract. 68, 618–627. 10.1111/ijcp.12365 24868587

[B85] Martinez-Calderon J. Flores-Cortes M. Morales-Asencio J. M. Luque-Suarez A. (2021). Intervention therapies to reduce pain-related fear in fibromyalgia syndrome: A systematic review of randomized clinical trials. Pain Med. 22, 481–498. 10.1093/pm/pnaa331 32989450

[B86] Meader N. King K. Llewellyn A. Norman G. Brown J. Rodgers M. (2014). A checklist designed to aid consistency and reproducibility of GRADE assessments: Development and pilot validation. Syst. Rev. 3, 82. 10.1186/2046-4053-3-82 25056145 PMC4124503

[B87] Mease P. J. Spaeth M. Clauw D. J. Arnold L. M. Bradley L. A. Russell I. J. (2011). Estimation of minimum clinically important difference for pain in fibromyalgia. Arthritis Care Res. Hob. 63, 821–826. 10.1002/acr.20449 21312349

[B88] Munguía-Izquierdo D. Legaz-Arrese A. (2007). Exercise in warm water decreases pain and improves cognitive function in middle-aged women with fibromyalgia. Clin. Exp. Rheumatol. 25, 823–830. 18173915

[B89] Murillo-Garcia A. Adsuar J. C. Villafaina S. Collado-Mateo D. Gusi N. (2022). Creative versus repetitive dance therapies to reduce the impact of fibromyalgia and pain: A systematic review and meta-analysis. Complement. Ther. Clin. Pract. 47, 101577. 10.1016/j.ctcp.2022.101577 35364519

[B90] Napadow V. Harris R. E. (2014). What has functional connectivity and chemical neuroimaging in fibromyalgia taught us about the mechanisms and management of “centralized” pain? Arthritis Res. Ther. 16, 1–8. 10.1186/S13075-014-0425-0/METRICS PMC4289059 25606591

[B91] Nüesch E. Häuser W. Bernardy K. Barth J. Jüni P. (2013). Comparative efficacy of pharmacological and non-pharmacological interventions in fibromyalgia syndrome: Network meta-analysis. Ann. Rheum. Dis. 72, 955–962. 10.1136/annrheumdis-2011-201249 22739992

[B92] Núñez M. Fernández-Solà J. Nuñez E. Fernández-Huerta J.-M. Godás-Sieso T. Gomez-Gil E. (2011). Health-related quality of life in patients with chronic fatigue syndrome: Group cognitive behavioural therapy and graded exercise versus usual treatment. A randomised controlled trial with 1 year of follow-up. Clin. Rheumatol. 30, 381–389. 10.1007/s10067-010-1677-y 21234629

[B93] Núñez-Fuentes D. Obrero-Gaitán E. Zagalaz-Anula N. Ibáñez-Vera A. J. Achalandabaso-Ochoa A. López-Ruiz M. (2021). Alteration of postural balance in patients with fibromyalgia syndrome—A systematic review and meta-analysis. Diagnostics 11, 127. 10.3390/diagnostics11010127 33467458 PMC7830486

[B94] O’Brien A. T. Deitos A. Triñanes Pego Y. Fregni F. Carrillo-de-la-Peña M. T. (2018). Defective endogenous pain modulation in fibromyalgia: A meta-analysis of temporal summation and conditioned pain modulation paradigms. J. Pain 19, 819–836. 10.1016/j.jpain.2018.01.010 29454976

[B95] Oliva V. Gregory R. Brooks J. C. W. Pickering A. E. (2022). Central pain modulatory mechanisms of attentional analgesia are preserved in fibromyalgia. Pain 163, 125–136. 10.1097/j.pain.0000000000002319 33941755 PMC8675057

[B96] Page M. J. McKenzie J. E. Bossuyt P. M. Boutron I. Hoffmann T. C. Mulrow C. D. (2021). The PRISMA 2020 statement: An updated guideline for reporting systematic reviews. BMJ 372, n71. 10.1136/bmj.n71 33782057 PMC8005924

[B97] Peinado-Rubia A. Osuna-Pérez M. C. Rodríguez-Almagro D. Zagalaz-Anula N. López-Ruiz M. C. Lomas-Vega R. (2020). Impaired balance in patients with fibromyalgia syndrome: Predictors of the impact of this disorder and balance confidence. Int. J. Environ. Res. Public Health 17, 3160. 10.3390/ijerph17093160 32370043 PMC7246608

[B98] Queiroz L. P. (2013). Worldwide epidemiology of fibromyalgia. Curr. Pain Headache Rep. 17, 356. 10.1007/s11916-013-0356-5 23801009

[B99] Redondo J. R. Justo C. M. Moraleda F. V. Velayos Y. G. Puche J. J. O. Zubero J. R. (2004). Long-term efficacy of therapy in patients with fibromyalgia: A physical exercise-based program and a cognitive-behavioral approach. Arthritis Care Res. Hob. 51, 184–192. 10.1002/art.20252 15077258

[B100] Richards S. C. M. Scott D. L. (2002). Prescribed exercise in people with fibromyalgia: Parallel group randomised controlled trial. BMJ 325, 185. 10.1136/bmj.325.7357.185 12142304 PMC117444

[B101] Ricoy-Cano A. J. Cortés-Pérez I. del Carmen Martín-Cano M. De La Fuente-Robles Y. M. (2021). Impact of fibromyalgia syndrome on female sexual function. *JCR J. Clin. Rheumatol.* Publ. Ah. 28, e574–e582. 10.1097/RHU.0000000000001758 34262004

[B102] Rooks D. S. Gautam S. Romeling M. Cross M. L. Stratigakis D. Evans B. (2007). Group exercise, education, and combination self-management in women with fibromyalgia. A randomized trial. Arch. Intern. Med. 167, 2192–2200. 10.1001/archinte.167.20.2192 17998491

[B103] Rothman K. J. Greenland S. Lash T. L. (2008). Modern epidemiology. Lippincott Williams and Wilkins.

[B104] Rücker G. Schwarzer G. (2020). Beyond the forest plot: The drapery plot. Res. Synth. Methods. 12, 13–19. 10.1002/jrsm.1410 32336044

[B105] Saeed S. A. Cunningham K. Bloch R. M. (2019). Depression and anxiety disorders: Benefits of exercise, yoga, and meditation. Am. Fam. Physician 99, 620–627. 31083878

[B106] Sañudo B. Carrasco L. de Hoyo M. Figueroa A. Saxton J. M. (2015). Vagal modulation and symptomatology following a 6-month aerobic exercise program for women with fibromyalgia. Clin. Exp. Rheumatol. 33, S41–S45. 25786042

[B107] Sañudo B. Galiano D. Carrasco L. de Hoyo M. McVeigh J. (2011). Effects of a prolonged exercise program on key health outcomes in women with fibromyalgia: A randomized controlled trial. J. Rehabil. Med. 43, 521–526. 10.2340/16501977-0814 21533333

[B108] Sañudo Corrales B. Galiano Orea D. Carrasco Páez L. Saxton J. de Hoyo Lora M. (2010). Respuesta autónoma e influencia sobre la calidad de vida de mujeres con fibromialgia tras una intervención de ejercicio físico a largo plazo. Rehabilitación 44, 244–249. 10.1016/j.rh.2009.11.008

[B109] Saracoglu I. Akin E. Aydin Dincer G. B. (2022). Efficacy of adding pain neuroscience education to a multimodal treatment in fibromyalgia: A systematic review and meta‐analysis. Int. J. Rheum. Dis. 25, 394–404. 10.1111/1756-185X.14293 35061337

[B110] Sarzi-Puttini P. Giorgi V. Marotto D. Atzeni F. (2020). Fibromyalgia: An update on clinical characteristics, aetiopathogenesis and treatment. Nat. Rev. Rheumatol. 16, 645–660. 10.1038/s41584-020-00506-w 33024295

[B111] Sauch Valmaña G. Vidal-Alaball J. Poch P. R. Peña J. M. Panadés Zafra R. Cantero Gómez F. X. (2020). Effects of a physical exercise program on patients affected with fibromyalgia. J. Prim. Care Community Health 11, 2150132720965071. 10.1177/2150132720965071 33084477 PMC7786411

[B112] Schachter C. L. Busch A. J. Peloso P. M. Sheppard M. S. (2003). Effects of short versus long bouts of aerobic exercise in sedentary women with fibromyalgia: A randomized controlled trial. Phys. Ther. 83, 340–358. Available at:. 10.1093/ptj/83.4.340 http://www.ncbi.nlm.nih.gov/pubmed/12665405 12665405

[B113] Schaefer C. Chandran A. Hufstader M. Baik R. McNett M. Goldenberg D. (2011). The comparative burden of mild, moderate and severe fibromyalgia: Results from a cross-sectional survey in the United States. Health Qual. Life Outcomes 9, 71. 10.1186/1477-7525-9-71 21859448 PMC3179696

[B114] Schmidt-Wilcke T. Diers M. (2017). New insights into the pathophysiology and treatment of fibromyalgia. Biomedicines 5, 22. 10.3390/biomedicines5020022 28536365 PMC5489808

[B115] Sencan S. Ak S. Karan A. Muslumanoglu L. Ozcan E. Berker E. (2004). A study to compare the therapeutic efficacy of aerobic exercise and paroxetine in fibromyalgia syndrome. J. Back Musculoskelet. Rehabil. 17, 57–61. 10.3233/bmr-2004-17204

[B116] Serrat M. Albajes K. Navarrete J. Almirall M. Lluch Girbés E. Neblett R. (2022). Effectiveness of two video-based multicomponent treatments for fibromyalgia: The added value of cognitive restructuring and mindfulness in a three-arm randomised controlled trial. Behav. Res. Ther. 158, 104188. 10.1016/j.brat.2022.104188 36116229

[B117] Serrat M. Almirall M. Musté M. Sanabria-Mazo J. P. Feliu-Soler A. Méndez-Ulrich J. L. (2020). Effectiveness of a multicomponent treatment for fibromyalgia based on pain neuroscience education, exercise therapy, psychological support, and nature exposure (NAT-FM): A pragmatic randomized controlled trial. J. Clin. Med. 9, 3348. 10.3390/jcm9103348 33081069 PMC7603188

[B118] Serrat M. Coll-Omaña M. Albajes K. Solé S. Almirall M. Luciano J. V. (2021a). Efficacy of the fibrowalk multicomponent program moved to a virtual setting for patients with fibromyalgia during the COVID-19 pandemic: A proof-of-concept rct performed alongside the state of alarm in Spain. Int. J. Environ. Res. Public Health 18, 10300. 10.3390/ijerph181910300 34639600 PMC8508552

[B119] Serrat M. Sanabria-Mazo J. P. Almirall M. Musté M. Feliu-Soler A. Méndez-Ulrich J. L. (2021b). Effectiveness of a multicomponent treatment based on pain neuroscience education, therapeutic exercise, cognitive behavioral therapy, and mindfulness in patients with fibromyalgia (fibrowalk study): A randomized controlled trial. Phys. Ther. 101, pzab200. 10.1093/ptj/pzab200 34499174

[B120] Silva H. J. de A. Assunção Júnior J. C. de Oliveira F. S. Oliveira J. M. de P. Figueiredo Dantas G. A. Lins C. A. de A. (2019). Sophrology versus resistance training for treatment of women with fibromyalgia: A randomized controlled trial. J. Bodyw. Mov. Ther. 23, 382–389. 10.1016/j.jbmt.2018.02.005 31103124

[B121] Skaer T. L. (2014). Fibromyalgia: Disease synopsis, medication cost effectiveness and economic burden. Pharmacoeconomics 32, 457–466. 10.1007/s40273-014-0137-y 24504852

[B122] Sosa-Reina M. D. Nunez-Nagy S. Gallego-Izquierdo T. Pecos-Martín D. Monserrat J. Álvarez-Mon M. (2017). Effectiveness of therapeutic exercise in fibromyalgia syndrome: A systematic review and meta-analysis of randomized clinical trials. Biomed. Res. Int. 2017, 2356346–2356414. 10.1155/2017/2356346 29291206 PMC5632473

[B123] Staud R. Koo E. Robinson M. E. Price D. D. (2007). Spatial summation of mechanically evoked muscle pain and painful aftersensations in normal subjects and fibromyalgia patients. Pain 130, 177–187. 10.1016/j.pain.2007.03.015 17459587 PMC2041939

[B124] Sterne J. A. C. Egger M. (2001). Funnel plots for detecting bias in meta-analysis: Guidelines on choice of axis. J. Clin. Epidemiol. 54, 1046–1055. 10.1016/S0895-4356(01)00377-8 11576817

[B125] Tan L. Cicuttini F. M. Fairley J. Romero L. Estee M. Hussain S. M. (2022). Does aerobic exercise effect pain sensitisation in individuals with musculoskeletal pain? A systematic review. BMC Musculoskelet. Disord. 23, 113. 10.1186/s12891-022-05047-9 35114987 PMC8815215

[B126] Tomas-Carus P. Biehl-Printes C. del Pozo-Cruz J. Parraca J. A. Folgado H. Pérez-Sousa M. Á. (2021). Effects of respiratory muscle training on respiratory efficiency and health-related quality of life in sedentary women with fibromyalgia: A randomised controlled trial. Clin. Exp. Rheumatol. 40, 1119–1126. 10.55563/clinexprheumatol/0v55nh 35748715

[B127] Tomas-Carus P. Branco J. C. Raimundo A. Parraca J. A. Batalha N. Biehl-Printes C. (2018). Breathing exercises must be a real and effective intervention to consider in women with fibromyalgia: A pilot randomized controlled trial. J. Altern. Complement. Med. 24, 825–832. 10.1089/acm.2017.0335 29653069

[B128] Tomas-Carus P. Gusi N. Hakkinen A. Hakkinen K. Raimundo A. Ortega-Alonso A. (2009). Improvements of muscle strength predicted benefits in HRQOL and postural balance in women with fibromyalgia: An 8-month randomized controlled trial. Rheumatology 48, 1147–1151. 10.1093/rheumatology/kep208 19605373

[B129] Tomas-Carus P. Hakkinen A. Gusi N. Leal A. Hakkinen K. Ortega-Alonso A. (2007). Aquatic training and detraining on fitness and quality of life in fibromyalgia. Med. Sci. Sport. Exerc. 39, 1044–1050. 10.1249/01.mss.0b0138059aec4 17596770

[B130] Vilarino G. T. Andreato L. V. de Souza L. C. Branco J. H. L. Andrade A. (2021). Effects of resistance training on the mental health of patients with fibromyalgia: A systematic review. Clin. Rheumatol. 40, 4417–4425. 10.1007/s10067-021-05738-z 33987785

[B131] Wigers S. H. Stiles T. C. Vogel P. A. (1996). Effects of aerobic exercise versus stress management treatment in fibromyalgia. A 4.5 year prospective study. Scand. J. Rheumatol. 25, 77–86. 10.3109/03009749609069212 8614771

[B132] Windthorst P. Mazurak N. Kuske M. Hipp A. Giel K. E. Enck P. (2017). Heart rate variability biofeedback therapy and graded exercise training in management of chronic fatigue syndrome: An exploratory pilot study. J. Psychosom. Res. 93, 6–13. 10.1016/j.jpsychores.2016.11.014 28107894

[B133] Wong A. Figueroa A. Sanchez-Gonzalez M. A. Son W.-M. Chernykh O. Park S.-Y. (2018). Effectiveness of tai chi on cardiac autonomic function and symptomatology in women with fibromyalgia: A randomized controlled trial. J. Aging Phys. Act. 26, 214–221. 10.1123/japa.2017-0038 28657825

[B134] Zijlstra T. R. van de Laar M. A. F. J. Bernelot Moens H. J. Taal E. Zakraoui L. Rasker J. J. (2005). Spa treatment for primary fibromyalgia syndrome: A combination of thalassotherapy, exercise and patient education improves symptoms and quality of life. Rheumatology 44, 539–546. 10.1093/rheumatology/keh537 15695301

