# Supplementary material for: Optimal dose and type of exercise to reduce pain, anxiety and increase quality of life in patients with fibromyalgia. A systematic review with meta-analysis
Source: Front Physiol. 2023 Apr 12;14:1170621. doi: 10.3389/fphys.2023.1170621 (PMC10130662; doi:10.3389/fphys.2023.1170621)
Supplement: Supplementary file 1 [file Table1.docx]

**Supplementary Table 1.** Characteristics of the studies included in the systematic review and meta-analysis

|  | | | **Experimental Group** | | | | | | | | **Control Group** | | | | | **Variables** | |
| --- | --- | --- | --- | --- | --- | --- | --- | --- | --- | --- | --- | --- | --- | --- | --- | --- | --- |
|  |  |  | **Sample Characteristics** | | | | **Intervention Characteristics** | | | | **Characteristics** | | | | | **Variable** | **Test** |
| **Study** | **Country** | **N** | **Ne** | **Age** | **BMI** | **% Fem** | **Type** | **Weeks** | **Sess/**  **week** | **Minutes** | **Nc** | **Age** | **BMI** | **% Fem** | **Type** |  |  |
| Andrade, CP et al 2019 | Brazil | 54 | 27 | 48 | NR | 100% | Circuit-based exercise | 16 | 2 | 45 | 27 | 47 | NR | 100% | Usual care | Pain  Impact  QoL  Anxiety | VAS  FIQ  SF-36  BAI |
| Arcos Carmona, IM et al 2011 | Spain | 53 | 27 | NR | NR | 100% | Circuit-based exercise | 10 | 2 | 60 | 26 | NR | NR | 100% | Magnetotherapy | QoL  Anxiety | SF-36  BAI |
| Arroyo-Fernández, R et al 2022 | Spain | 80 | 40 | 50.6 | 27.2 | 95% | Circuit-based exercise | 2 | 3 | 20 | 40 | 50.8 | 27.4 | 90% | Usual care | Pain  Impact  Anxiety | VAS  FIQ  STAI |
| Assumpcao, A et al 2018 | Brazil | 46 | 16 | 45.7 | 28.1 | 100% | Circuit-based exercise | 12 | 2 | 40 | 16 | 46.9 | 29.4 | 100% | Usual care | Pain  Impact  QoL  Anxiety | VAS  FIQ  SF-36  FIQ |
|  |  |  | 14 | 47.9 | 28.9 | 100% | Circuit-based exercise | 12 | 2 | 40 |  |  |  |  |  |  |  |
| Astin, JA et al 2003 | United States | 128 | 64 | NR | NR | 100% | Exercise-movement | 8 | 1 | 150 | 64 | NR | NR | 100% | Education | Impact | FIQ |
| Atan, T et al 2020 | Turkey | 55 | 19 | 46.6 | NR | 100% | Circuit-based exercise | 6 | 5 | 35 | 17 | 52.7 | NR | 100% | Usual care | Pain  Impact  QoL | VAS  FIQ  SF-36 |
|  |  |  | 19 | 47.4 | NR | 100% | Circuit-based exercise | 6 | 5 | 55 |  |  |  |  |  |  |  |
| Baptista, AS et al 2012 | Brazil | 80 | 40 | 49.5 | NR | 100% | Exercise-movement | 16 | 2 | 60 | 40 | 49.1 | NR | 100% | Usual care | Pain  Impact  QoL  Anxiety | VAS  FIQ  SF-36  STAI |
| Carson, JW et al 2010 | United States | 50 | 22 | 51.4 | NR | 100% | Exercise-movement | 8 | 1 | 120 | 28 | 55.8 | NR | 100% | Usual care | Pain  Impact  Anxiety | FIQ-R  FIQ  FIQ |
| Castel, A et al 2013 | Spain | 155 | 81 | 49 | 27.6 | 100% | Exercise-movement | 12 | 2 | 60 | 74 | 48.8 | 28.8 | 100% | Usual care | Pain  Impact | VAS  FIQ |
| Cedraschi, C et al 2004 | Switzerland | 164 | 84 | NR | NR | 92.8% | Circuit-based exercise | 6 | 2 | 90 | 80 | NR | NR | 92.5% | Usual care | Pain  Impact  QoL  Anxiety | FIQ  FIQ  SF-36  PGWE |
| Chan, JSM et al 2014 | China | 150 | 75 | 39.1 | 22.3 | 61.3% | Exercise-movement | 9 | 2 | 90 | 75 | 38.9 | 21.6 | 82.7% | Usual care | Anxiety | HADS |
| Chan, JSM et al 2017 | China | 108 | 46 | 39.5 | 20.8 | 100% | Exercise-movement | 9 | 2 | 90 | 62 | 42 | 20.1 | 100% | Usual care | Anxiety | HADS |
| Clarke-Jenssen, A et al 2014 | Norway | 129 | 43 | 46 | 29 | 86.1% | Circuit-based exercise | 4 | 5 | 90 | 44 | 45 | 28 | 95.5% | Usual care | Pain | VAS |
|  |  |  | 42 | 46 | 27 | 95.2% | Circuit-based exercise | 4 | 5 | 90 |  |  |  |  |  |  |  |
| Da Costa, D et al 2005 | Canada | 79 | 39 | 48.2 | 28 | 100% | Circuit-based exercise | 12 | NR | NR | 40 | 52.3 | 28.1 | 100% | Usual care | Impact | FIQ |
| Ekici, G et al 2017 | Turkey | 36 | 15 | 37.1 | 23.2 | 100% | Exercise-movement | 4 | 3 | 60 | 21 | 36.86 | 22.48 | 100% | Usual care | Pain  Impact  Anxiety | FIQ  FIQ  STAI |
| Ericsson, A et al 2016 | Sweden | 130 | 67 | NR | NR | 100% | Circuit-based exercise | 15 | 2 | 60 | 63 | NR | NR | 100% | Relaxation | Pain  Anxiety | PCS  HADS |
| Espí-López, G et al 2016 | Spain | 35 | 13 | 53.1 | 27 | 92.3% | Circuit-based exercise | 8 | 2 | 60 | 9 | 57.1 | 26.3 | 100% | Usual care | Pain  Impact | VAS  FIQ |
|  |  |  | 13 | 51.2 | 26.4 | 92.3% | Circuit-based exercise | 8 | 2 | 60 |  |  |  |  |  |  |  |
| Fonseca, ACS et al 2021 | Brazil | 46 | 27 | 53.8 | 27.2 | 100% | Circuit-based exercise | 9 | 1 | 60 | 19 | 54.4 | 29.4 | 100% | Education | Pain  Impact  Anxiety | VAS  FIQ  BAI |
| Fontaine, KR et al 2007 | United States | 48 | 22 | 48 | 31.6 | NR | Circuit-based exercise | 12 | 1 | 90 | 26 | 52 | 31.2 | NR | Education | Pain  Impact | VAS  FIQ |
| Fontaine, KR et al 2010 | United States | 84 | 46 | 46.4 | 31.4 | 93.5% | Circuit-based exercise | 12 | 1 | 90 | 38 | 49 | 29.8 | 100% | Education | Pain  Impact | VAS  FIQ |
| García-Martínez, AM et al 2012 | Spain | 28 | 14 | 59.3 | 27.6 | 100% | Circuit-based exercise | 12 | 3 | 60 | 14 | 58.6 | 29 | 100% | Usual care | Impact  QoL | FIQ  SF-36 |
| Garrido-Ardila, EM et al 2020 | Spain | 69 | 36 | 56.1 | NR | 100% | Exercise-movement | 6 | 2 | 30 | 33 | 54.4 | NR | 100% | Usual care | Impact | FIQ |
|  |  |  |  |  |  |  |  |  |  |  | 34 | 56.2 | NR | 100% | Acupuncture |  |  |
| Giannotti, E et al 2014 | Italy | 32 | 20 | 52.8 | 24.3 | 95% | Circuit-based exercise | 10 | 2 | 60 | 12 | 51.3 | 23.4 | 91.7% | Usual care | Pain  Impact | VAS  FIQ |
| Gowans, SE et al 2001 | Canada | 31 | 15 | 46.7 | NR | 86.7% | Circuit-based exercise | 23 | 3 | 30 | 16 | 49.1 | NR | 93.8% | Usual care | Impact  Anxiety | FIQ  STAI |
| Gunendi, Z et al 2008 | Turkey | 32 | 17 | 45 | 23.8 | 100% | Circuit-based exercise | 4 | 5 | 30 | 15 | 43 | 26.2 | 100% | Usual care | Pain  Anxiety | VAS  HADS |
| Gusi, N et al 2006 | Spain | 34 | 17 | 51 | 27 | 100% | Circuit-based exercise | 12 | 3 | 60 | 17 | 51 | 27 | 100% | Usual care | Pain | VAS |
| Hammond, A et al 2006 | United Kingdom | 133 | 71 | 48.4 | NR | 88.7% | Exercise-movement | 10 | 5 | 30 | 62 | 48.7 | NR | 91.9% | Relaxation | Pain  Impact  Anxiety | FIQ  FIQ  FIQ |
| Haugmark, T et al 2021 | Norway | 170 | 85 | 41 | NR | 92% | Circuit-based exercise | NR | NR | NR | 84 | 44 | NR | 95% | Usual care | Pain | NPRS |
| Hernando-Garijo, I et al 2021 | Spain | 34 | 17 | 51.8 | 27.3 | 100% | Circuit-based exercise | 15 | 2 | 50 | 17 | 5516 | 25.9 | 100% | Usual care | Pain  Impact  Anxiety | VAS  FIQ  HADS |
| Izquierdo-Alventosa, R et al 2020 | Spain | 32 | 16 | 53.1 | 27.8 | 100% | Circuit-based exercise | 8 | 2 | 60 | 16 | 55.1 | 28.2 | 100% | Usual care | Pain  Impact  Anxiety | VAS  FIQ  HADS |
| Izquierdo-Alventosa, R et al 2021 | Spain | 32 | 16 | 53.1 | 27.8 | 100% | Circuit-based exercise | 8 | 2 | 60 | 16 | 55.1 | 28.2 | 100% | Usual care | Pain  Impact | VAS  FIQ-R |
| Jones, KD et al 2012 | United States | 98 | 51 | 53.3 | 30.9 | 92.2% | Exercise-movement | 12 | 2 | 90 | 47 | 54.8 | 30.1 | 93.6% | Education | Pain  Impact | BPI  FIQ |
| Kashikar-Zuck, S et al 2018 | United States | 36 | 17 | NR | NR | NR | Circuit-based exercise | 8 | 2 | 90 | 19 | NR | NR | NR | Psychological treatment | Pain | VAS |
| Kayo, AH et al 2012 | Brazil | 90 | 30 | 47.7 | 26.3 | 100% | Circuit-based exercise | 16 | 3 | 60 | 30 | 46.1 | 26.7 | 100% | Usual care | Pain  Impact | VAS  FIQ |
|  |  |  | 30 | 46.7 | 26.2 | 100% | Circuit-based exercise | 16 | 3 | 60 |  |  |  |  |  |  |  |
| King, SJ et al 2002 | Canada | 152 | 35 | 47.4 | 32 | 100% | Circuit-based exercise | 12 | 2 | 40 | 34 | 47.3 | 29.6 | 100% | Usual care | Impact | FIQ |
|  |  |  | 42 | 45.2 | 28.2 | 100% | Circuit-based exercise | 12 | 2 | 40 |  |  |  |  |  |  |  |
| Kingsley, JD et al 2005 | United States | 29 | 15 | 45 | 30.3 | 100% | Circuit-based exercise | 12 | 2 | 30 | 14 | 47 | 32 | 100% | Usual care | Impact | FIQ |
| Kurt, EE et al 2016(Kurt, 2016) | Turkey | 109 | 36 | 35.13 | NR | 100% | Circuit-based exercise | 3 | 5 | 35 | 37 | 38.1 | NR | 100% | Balneotherapy | Impact | FIQ |
|  |  |  | 36 | 41.9 | NR | 100% | Circuit-based exercise | 3 | 5 | 35 |  |  |  |  |  |  |  |
| Larsson, A et al 2015 | Sweden | 130 | 67 | 50.81 | 27.39 | 100% | Circuit-based exercise | 15 | 2 | 60 | 63 | 52.1 | 28.6 | 100% | Relaxation | Pain  Impact | VAS  FIQ |
| Latorre-Román, PA et al 2015 | Spain | 36 | 20 | 51.7 | 26.2 | 100% | Circuit-based exercise | 18 | 3 | 60 | 16 | 50.3 | 26.5 | 100% | Usual care | Pain  Impact | VAS  FIQ |
| Lorena, SB et al 2022 | Brazil | 40 | 19 | 46.5 | NR | 100% | Circuit-based exercise | 10 | 1 | 90 | 21 | 46.4 | NR | 100% | Usual care | Pain  Impact | VAS  FIQ |
| Maddali-Bongi, S et al 2016 | Italy | 44 | 22 | 50.4 | NR | 100% | Exercise-movement | 16 | 2 | 60 | 22 | 54.3 | NR | 100% | Eduaction | Impact  QoL  Anxiety | FIQ  SF-36  HADS |
| Mannerkorpi, K et al 2000 | Sweden | 48 | 28 | 45 | NR | 100% | Circuit-based exercise | 24 | 1 | 35 | 20 | 47 | NR | 100% | Education | Pain  Impact  QoL  Anxiety | FIQ  FIQ  SF-36  AIMS |
| Mannerkorpi, K et al 2009 | Sweden | 133 | 69 | 46.6 | NR | 100% | Circuit-based exercise | 20 | 1 | 45 | 64 | 46.5 | NR | 100% | Education | Pain  Impact  QoL  Anxiety | FIQ  FIQ  SF-36  HADS |
| Martín, J et al 2014 | Spain | 110 | 54 | 48.7 |  | 90.7% | Circuit-based exercise | 6 | 2 | 45 | 56 | 51.6 | NR | 91.1% | Usual care | Impact  Anxiety | FIQ  HADS |
| Munguía-Izquierdo, D et al 2007 | Spain | 53 | 29 | 50 | 27 | 100% | Circuit-based exercise | 16 | 3 | 70 | 24 | 46 | 27 | 100% | Usual care | Pain  Impact | FIQ  FIQ |
| Núñez, M et al 2011 | Spain | 115 | 58 | 42.65 | NR | 89.7% | Circuit-based exercise | 12 | 3 | 40 | 57 | 44.3 | NR | 82.5% | Usual care | QoL | SF-36 |
| Redondo, JR et al 2004 | Spain | 40 | 19 | NR | NR | 100% | Circuit-based exercise | 8 | 5 | 45 | 21 | NR | NR | 100 % | Psychology | Pain  Impact  QoL  Anxiety | FIQ  FIQ  SF-36  BAI |
| Richards, SCM et al 2002 | United Kingdom | 133 | 65 | 48 | NR | 89.9% | Circuit-based exercise | 12 | 2 | 50 | 68 | 45 | NR | 95.5% | Relaxation | Impact | FIQ |
| Rooks, DS et al 2007 | United States | 135 | 35 | 48 | 29 | 100% | Circuit-based exercise | 16 | 2 | 60 | 27 | 51 | 29 | 100% | Education | Pain  Impact  QoL  Anxiety | FIQ  FIQ  SF-36  FIQ |
|  |  |  | 35 | 50 | 30 | 100% | Circuit-based exercise | 16 | 2 | 60 |  |  |  |  |  |  |  |
|  |  |  | 38 | 50 | 29 | 100% | Circuit-based exercise | 16 | 2 | 60 |  |  |  |  |  |  |  |
| Sañudo-Corrales, B et al 2010 | Spain | 38 | 18 | 55.9 | 29.2 | 100% | Circuit-based exercise | 20 | 2 | 55 | 20 | 56.6 | 29.8 | 100% | Usual care | QoL | SF-36 |
| Sañudo-Corrales, B et al 2011 | Spain | 38 | 18 | 55.5 | 28.2 | 100% | Circuit-based exercise | 24 | 2 | 55 | 20 | 56.2 | 29.1 | 100% | Usual care | Impact  QoL | FIQ  SF-36 |
| Sañudo-Corrales, B et al 2015 | Spain | 28 | 16 | 55 | 29.6 | 100% | Circuit-based exercise | 24 | 2 | 60 | 12 | 58 | 29.7 | 100% | Usual care | Pain  Anxiety | VAS  VAS |
| Sauch-Valmaña, G et al 2020 | Spain | 48 | 25 | 55 | NR | 100% | Circuit-based exercise | 12 | 2 | 90 | 23 | 52.9 | NR | 100% | Usual care | Pain  Impact  QoL | VAS  FIQ  SF-36 |
| Schachter, CL et al 2003 | Canada | 143 | 51 | 41.3 | NR | 100% | Circuit-based exercise | 16 | 5 | 30 | 36 | 42.5 | NR | 100% | Usual care | Pain  Impact  Anxiety | VAS  FIQ  FIQ |
|  |  |  | 56 | 41.9 | NR | 100% | Circuit-based exercise | 16 | 5 | 15 |  |  |  |  |  |  |  |
| Sencan, S et al 2004 | Turkey | 60 | 20 | 35.4 | 24.2 | 100% | Circuit-based exercise | 6 | 3 | 40 | 20 | 35.6 | 24.6 | 100% | Electrotherapy | Pain | VAS |
|  |  |  |  |  |  |  |  |  |  |  | 20 | 32.5 | 24.3 | 100% | Drugs |  |  |
| Serrat, M et al 2020 | Spain | 169 | 84 | 54.1 | 27.7 | 97.6% | Circuit-based exercise | 12 | 1 | 40 | 85 | 53.2 | 26.8 | 100% | Usual care | Pain  Impact  Anxiety | PRI  FIQ  HADS |
| Serrat, M et al 2021a | Spain | 272 | 135 | 53.4 | 27.9 | 97% | Circuit-based exercise | 12 | 1 | 60 | 137 | 53.2 | 26.1 | 100% | Usual care | Pain  Impact  QoL  Anxiety | VAS  FIQ-R  SF-36  HADS |
| Serrat, M et al 2021b | Spain | 151 | 75 | 54.9 | 27.4 | 94.7% | Circuit-based exercise | 12 | 1 | 60 | 76 | 53.8 | 26.8 | 92.1% | Usual care | Impact  QoL  Anxiety | FIQ-R  SF-36  HADS |
| Serrat, M et al 2022 | Spain | 210 | 107 | 52.5 | 26.9 | 97.3% | Circuit-based exercise | 12 | 1 | 60 | 103 | 53.5 | 27.6 | 96.7% | Usual care | Pain  Impact  QoL  Anxiety | VAS  FIQ-R  SF-36  HADS |
| Silva, HJ de A et al 2019 | Brazil | 60 | 30 | 44.9 | 26.4 | 100% | Circuit-based exercise | 12 | 2 | 40 | 30 | 49.4 | 26.6 | 100% | Relaxation | QoL | SF-36 |
| Tomas-Carus, P et al 2007 | Portugal | 34 | 17 | 51 | 27 | 100% | Circuit-based exercise | 12 | 3 | 60 | 17 | 51 | 27 | 100% | Usual care | Impact  QoL | FIQ  SF-36 |
| Tomas-Carus, P et al 2009 | Portugal | 30 | 15 | 50.7 | 28.8 | 100% | Circuit-based exercise | 32 | 3 | 60 | 15 | 50.9 | 26.6 | 100% | Usual care | QoL | SF-36 |
| Tomas-Carus, P et al 2018 | Portugal | 35 | 18 | 54.1 | 25.1 | 100% | Exercise-movement | 21 | 7 | 30 | 17 | 50.8 | 27.9 | 100% | Usual care | Pain  Impact  Anxiety | FIQ  FIQ  FIQ |
| Tomas-Carus, P et al 2022 | Portugal | 35 | 18 | 54.1 | 25.1 | 100% | Exercise-movement | 18 | 3 | 30 | 17 | 50.8 | 27.9 | 100% | Usual care | QoL | SF-36 |
| Wigers, SH et al 1996 | Norway | 60 | 20 | 43 | NR | 90% | Circuit-based exercise | 17 | 3 | 45 | 20 | 44 | NR | 90% | Usual care | Pain | VAS |
|  |  |  |  |  |  |  |  |  |  |  | 20 | 46 | NR | 90% | Psychology |  |  |
| Windthorst, P et al 2017 | Germany | 24 | 11 | 50 | 25.5 | 100% | Circuit-based exercise | 12 | 2 | 50 | 13 | 51.4 | 25.6 | 100% | Relaxation | QoL | SF-36 |
| Wong, A et al 2018 | United States | 37 | 18 | 51 | 23.1 | 100% | Circuit-based exercise | 18 | 2 | 55 | 19 | 51 | 22.2 | 100% | Usual care | Pain | VAS |
| Zijlstra, TR et al 2005 | Netherlands | 134 | 58 | 48 | NR | 94.8% | Circuit-based exercise | 2 | 2 | 60 | 76 | 47 | NR | 96.1% | Usual care | Pain  Impact  Anxiety | FIQ  FIQ  FIQ |

**Abbreviations:** K, Number of comparisons provided by this study; N, Total sample size; Ne, Participants in experimental Intervention; BMI, Body Mass Index; % Fem, Percentage of women; Sess, sessions; Nc, Participants in control intervention; QoL, Quality of life; VAS, Visual Analogue Scale; NPRS, Numeric Pain Rating Scale; FIQ, Fibromyalgia Impact Questionnaire; BAI, Beck Anxiety Inventory; PGWB, Psychological General Well-Being; STAI, State Trait Anxiety Inventory; HADS, Hospital Anxiety and Depression State. BPI, Brief Pain Inventory; AIMS, Arthritis Impact Measurement Scales
