# Supplementary material for: Optimal dose and type of exercise to reduce pain, anxiety and increase quality of life in patients with fibromyalgia. A systematic review with meta-analysis
Source: Front Physiol. 2023 Apr 12;14:1170621. doi: 10.3389/fphys.2023.1170621 (PMC10130662; doi:10.3389/fphys.2023.1170621)
Supplement: Supplementary file 2 [file DataSheet1.docx]

Supplementary Material

**Optimal dose and type of exercise to reduce pain, anxiety and increase quality of life in patients with fibromyalgia. A systematic review with meta-analysis**

**Daniel Rodríguez-Almagro, PT, PhD,^1^ María Del Moral-García, PT, MSc,^2^ María del Carmen López-Ruiz, PT, PhD,^2^ Irene Cortés-Pérez, PT, PhD,^2^ Esteban Obrero-Gaitán, PT, PhD,^2,^* Rafael Lomas-Vega, PT, PhD^2^**

*** Correspondence:** Esteban Obrero-Gaitán; [eobrero@ujaen.es](mailto:eobrero@ujaen.es)

# Supplementary Figures and Tables

## Supplementary Figures

**Figure S1. Funnel** Plot of the immediate effect of PEBT on pain

**Figure S2.** Funnel Plot of the immediate effect of PEBT on FMS impact

**Figure S3.** Funnel Plot of the immediate effect of PEBT on QoL-physical dimension

**Figure S4.** Funnel Plot of the immediate effect of PEBT on QoL-mental dimension

**Figure S5.** Funnel Plot of the immediate effect of PEBT on anxiety

## Supplementary Tables

**Table S1.** Search strategy in each database

| **DATABASES** | **SEARCH STRATEGY** |
| --- | --- |
| **PubMed Medline** | (fatigue syndrome, chronic[mh] OR fatigue syndrome, chronic[tiab] OR fibromyalgia[mh] OR fibromyalgia[tiab]) AND (exercise[mh] OR exercise[tiab] OR exercise therapy[mh] OR exercise therapy[tiab] OR physical exercise[tiab] OR physical activity[tiab] OR training[tiab]) AND (randomized controlled trial[publication type] OR randomized controlled trial[tiab] OR clinical trial[publication type] OR clinical trial[tiab] OR controlled clinical trial[publication type] OR controlled clinical trial[tiab]) NOT (systematic review[publication type] OR systematic review[tiab] OR meta-analysis[publication type] OR metaanalysis[tiab] OR review[publication type] OR review[tiab]) |
| **Scopus** | (TITLE-ABS-KEY ("chronic fatigue syndrome" OR "fibromyalgia" OR "fibromyalgia syndrome") AND TITLE-ABS-KEY ("exercise" OR "exercise" OR "physical exercise" OR "physical activity" OR "exercise therapy" OR "training") AND TITLE-ABS-KEY ("randomized controlled trial" OR "clinical trial"OR"controlled clinical trial") NOT TITLE-ABS-KEY ("systematic review " OR "metaanalysis"OR"review")) |
| **Web of Science** | (TS=(*chronic fatigue syndrome* OR *fibromyalgia* OR *fibromyalgia syndrome*) AND TS=(*exercise* OR *physical exercise* OR *physical activity* OR *exercise therapy* OR *training*)) AND TS=(*randomized controlled trial* OR *clinical trial* OR *controlled clinical trial*) NOT TS=(*systematic review* OR *metaanalysis* OR *review*) |
| **CINAHL Complete** | AB ("chronic fatigue syndrome" OR fibromyalgia) AND AB (exercise OR "exercise therapy" OR "physical activity" OR training) AND AB ("randomized controlled trial" OR "clinical trial" OR "controlled clinical trial") NOT AB ("systematic review " OR "metaanalysis" OR "review") |
| **PEDro** | Fibromyalgia AND exercise |

**Table S2.** Results in subgroup analysis according to follow time assessment and specific types of PEBT

| **Variables** | **Subgroups by specific type of PEBT** | | **Subgroups by follow-up assessment** | | |
| --- | --- | --- | --- | --- | --- |
|  | **Circuit-based exercise** | **Exercise movement techniques** | **Up to 12 weeks** | **Up to 24 weeks** | **Up to 48 weeks** |
| **Pain** | *k* = 45; SMD -0.55; 95% CI -0.72 to -0.38; *p*<0.001 | *k* = 8; SMD -1.1; 95% CI -1.48 to -0.63; *p*<0.001 | *k* = 7; SMD -0.74; 95% CI -1.03 to -0.45; *p*<0.001 | *k* = 11; SMD -0.19; 95% CI -0.32 to -0.06; *p*=0.004 | *k* = 6; SMD -0.04; 95% CI -0.32 to 0.24; *p*=0.78 |
| **FMS Impact** | *k* = 45; SMD -0.54; 95% CI -0.71 to -0.38; *p*<0.001 | *k* = 8; SMD -0.32; 95% CI -0.48 to -0.15; *p*=0.001 | *k* = 8; SMD -0.51; 95% CI -0.84 to -0.18; *p*=0.003 | *k* = 10; SMD -0.27; 95% CI -0.41 to -0.15; *p*<0.001 | *k* = 6; SMD -0.3; 95% CI -0.45 to -0.15; *p*<0.001 |
| **QoL-Physical** | *k* = 25; SMD 0.5; 95% CI 0.32 to 0.69; *p*<0.001 | *k* = 2; SMD 0.59; 95% CI -0.11 to 1.28; *p*=0.096 | NP | *k* = 4; SMD 0.21; 95% CI -0.16 to 0.58; *p*=0.26 | *k* = 45; SMD 0.07; 95% CI -0.25 to 0.39; *p*=0.67 |
| **QoL-Mental** | *k* = 21; SMD 0.54; 95% CI 0.36 to 0.72; *p*<0.001 | *k* = 2; SMD -0.32; 95% CI -0.92 to 0.28; *p*=0.3 | NP | *k* = 4; SMD 0.23; 95% CI -0.13 to 0.6; *p*=0.21 | *k* = 3; SMD -0.07; 95% CI -0.32 to 0.3; *p*=0.96 |
| **Anxiety** | *k* = 25; SMD -0.37; 95% CI -0.5 to -0.24; *p*<0.001 | *k* = 5; SMD -0.37; 95% CI -0.66 to -0.08; *p*=0.013 | *k* = 4; SMD -0.24; 95% CI -0.41 to -0.07; *p*=0.007 | *k* = 6; SMD 0.01; 95% CI -0.22 to 0.24; *p*=0.93 | *k* = 4; SMD -0.004; 95% CI -0.28 to 0.27; *p*=0.977 |

Abbreviations: PEBT, Physical Exerise-Based Therapy; FMS, Fibromyalgia Syndrome; QOL, Quality of Life; SMD, Standardized Mean Difference; 95% CI, 95% Confidence Interval; *p*, p-value; NP, Not possible to calculate.

**Table S3.** Results in subgroup analysis to estimate the most optimal dose of PEBT

| **Outcome** | **Dose** | | **SMD** | **95% CI** | ***P*** |
| --- | --- | --- | --- | --- | --- |
| **Pain** | **Total number of sessions** | <20 | -0.59 | -0.84 to -0.33 | <0.001 |
|  |  | 21-40 | -0.83 | -1.1 to -0.56 | <0.001 |
|  |  | 41-60 | -0.42 | -0.95 to 0.12 | 0.111 |
|  |  | >60 | -0.11 | -0.76 to 0.53 | 0.734 |
|  | **Sessions per week** | 1 | -0.76 | -0.88 to -0.64 | <0.001 |
|  |  | 2 | -0.45 | -0.72 to -0.18 | 0.001 |
|  |  | 3 | -0.82 | -1.2 to -0.48 | <0.001 |
|  |  | 4 | NP | NP | NP |
|  |  | 5 | -0.39 | -0.62 to -0.16 | 0.001 |
|  | **Duration of each session in minutes** | 0-30 | -0.36 | -0.84 to 0.12 | 0.14 |
|  |  | 31-60 | -0.58 | -0.79 to -0.38 | <0.001 |
|  |  | 61-90 | -1.08 | -1.55 to -0.62 | <0.001 |
|  |  | 91-120 | NP | NP | NP |
| **FMS Impact** | **Total number of sessions** | <20 | -0.41 | -0.66 to -0.17 | 0.001 |
|  |  | 21-40 | -0.63 | -0.87 to -0.35 | <0.001 |
|  |  | 41-60 | -0.44 | -1.14 to 0.25 | 0.21 |
|  |  | >60 | -0.33 | -1.1 to 0.35 | 0.336 |
|  | **Sessions per week** | 1 | -0.43 | -0.74 to -0.12 | 0.007 |
|  |  | 2 | -0.38 | -0.62 to -0.15 | 0.001 |
|  |  | 3 | -0.57 | -1.03 to -0.12 | 0.013 |
|  |  | 4 | NP | NP | NP |
|  |  | 5 | -0.43 | -0.88 to 0.01 | 0.054 |
|  | **Duration of each session in minutes** | 0-30 | -0.2 | -0.67 to 0.27 | 0.4 |
|  |  | 31-60 | -0.5 | -0.7 to -0.3 | <0.001 |
|  |  | 61-90 | -0.31 | -0.66 to 0.27 | 0.089 |
|  |  | 91-120 | -0.37 | -1.17 to 0.5 | 0.363 |
| **QoL-Physical** | **Total number of sessions** | 0-20 | 0.38 | 0.05 to 0.7 | 0.023 |
|  |  | 21-40 | 0.57 | 0.32 to 0.79 | <0.001 |
|  |  | 41-60 | NP | NP | NP |
|  |  | > 60 | NP | NP | NP |
|  | **Sessions per week** | 1 | 0.57 | 0.22 to 0.91 | 0.001 |
|  |  | 2 | 0.35 | 0.12 to 0.58 | 0.003 |
|  |  | 3 | 0.75 | 0.24 to 1.24 | 0.003 |
|  |  | 4 | NP | NP | NP |
|  |  | 5 | NP | NP | NP |
|  | **Duration of each session in minutes** | 0-30 | NP | NP | NP |
|  |  | 31-60 | 0.55 | 0.37 to 0.74 | <0.001 |
|  |  | 61-90 | NP | NP | NP |
|  |  | 91-120 | 0.09 | -0.34 to 0.52 | 0.7 |
| **QoL-Mental** | **Total number of sessions** | 0-20 | 0.18 | -0.27 to 0.63 | 0.43 |
|  |  | 21-40 | 0.51 | 0.28 to 0.73 | <0.001 |
|  |  | 41-60 | NP | NP | NP |
|  |  | > 60 | NP | NP | NP |
|  | **Sessions per week** | 1 | 0.35 | -0.1 to 0.81 | 0.13 |
|  |  | 2 | 0.35 | 0.11 to 0.58 | 0.003 |
|  |  | 3 | 0.62 | 0.14 to 1.04 | 0.011 |
|  |  | 4 | NP | NP | NP |
|  |  | 5 | 1.1 | 0.55 to 1.63 | <0.001 |
|  | **Duration of each session in minutes** | 0-30 | NP | NP | NP |
|  |  | 31-60 | 0.51 | 0.31 to 0.71 | <0.001 |
|  |  | 61-90 | NP | NP | NP |
|  |  | 91-120 | NP | NP | NP |
| **Anxiety** | **Total number of sessions** | 0-20 | -0.45 | -0.62 to -0.3 | <0.001 |
|  |  | 21-40 | -0.21 | -0.39 to -0.05 | 0.013 |
|  |  | 41-60 | NP | NP | NP |
|  |  | > 60 | -0.36 | -0.75 to 0.02 | 0.063 |
|  | **Sessions per week** | 1 | -0.41 | -0.62 to -0.21 | <0.001 |
|  |  | 2 | -0.33 | -0.51 to -0.14 | 0.001 |
|  |  | 3 | -0.73 | -1.16 to -0.3 | 0.001 |
|  |  | 4 | NP | NP | NP |
|  |  | 5 | -0.19 | -0.84 to 0.28 | 0.32 |
|  | **Duration of each session in minutes** | 0-30 | -0.27 | -0.61 to 0.07 | 0.12 |
|  |  | 31-60 | -0.4 | -0.51 to -0.3 | <0.001 |
|  |  | 61-90 | -0.34 | -0.73 to -0.07 | 0.021 |
|  |  | 91-120 | NP | NP | NP |

Abbreviations: SMD, Standardized Mean Difference; 95% CI, 95% Confidence Interval; P, P-value; NP, Not possible to calculate
